# Discovery of new pyridine 3-carboxylic acid-based pharmacophores as dual anti-inflammatory and anti-hyperglycemic agents

**DOI:** 10.1038/s41598-025-17841-1

**Published:** 2025-10-03

**Authors:** K. Ramakrishnan, Lenin Nachimuthu, Reshma Rajan, J. Premkumar, Vallabh Mulay, S. Meenakshi, Chandrakala A. Narasimhulu, Pragney Deme, Sanjay Rajagopalan, Ramanathan Lalgudi, Akella Sivaramakrishna, S. Karthikeyan, Rajagopal Desikan

**Affiliations:** 1 Department of Chemistry, School of Advanced Sciences, Vellore Institute of Technology, Vellore, 632014 Tamilnadu India; 2 Department of English, School of Social Sciences and Languages, Vellore Institute of Technology, Vellore, 632014 Tamilnadu India; 3 Division of Metabolic and Cardiovascular Sciences, Burnett School of Biomedical Sciences, University Of Central Florida, Orlando, USA; 4 Department of Neurology, The Johns Hopkins University School of Medicine, Baltimore, MD USA; 5 Department of Medicine, School of Medicine, Case Western Reserve University, Cleveland, OH USA; 6 Aries Science & Technology, Columbus, OH USA

**Keywords:** Anti-hyperglycemic activity, Α-amylase, Nicotinic acid, Anti-inflammatory, Anti-oxidant, Biochemistry, Drug discovery, Molecular biology, Chemistry

## Abstract

Novel nicotinic acid derivatives were synthesized as potent anti-diabetic agents. The developed inhibitors were investigated for their pharmacological in vitro screening, such as anti-diabetic, anti-inflammation, and in silico analysis. It has good interactions with α-amylase protein, with a range of -7.18 to -5.55 kcal/mol. The inhibitors were synthesized by employing the Steglich-type reaction to couple alcohol and amine derivatives. These molecules were characterized using NMR, FT-IR, and Mass spectrometry techniques. Anti-hyperglycemic activity has been screened using α-amylase protein, and the half-maximal inhibition efficiency (IC_50_) value was evaluated, ranging from 1.324 ± 0.17 to 1.516 ± 0.14 µg. The positive control (acarbose) range is 1.273 ± 0.12 µg. Induced human RBC hemolysis assay was utilized for anti-inflammatory activity, and the cumulative observation results were compared with the reference drug (ketorolac- IC_50_ = 11.79 ± 0.17 µM), with IC_50_ values between 14.06 ± 0.15 to 85.56 ± 0.25 µM. Moreover, the antioxidant ability of synthesized inhibitor compounds was evaluated by radical scavenging methods. The efficacy of inhibitors **2b** and **2 h** (IC_50_ = 15.63 ± 0.13 µM and 12.88 ± 0.19 µM (DPPH), IC_50_ = 19.89 ± 0.25 µM and 16.35 ± 0.25 µM (ABTS)) exhibited good results against DPPH and ABTS radicals with ascorbic acid(standard) (DPPH: IC_50_ = 11.81 ± 0.04 µM and ABTS: 11.90 ± 0.01 µM). The synthesized derivatives show promising results of anti-inflammatory and anti-hyperglycemic properties. This provides a path to developing the new therapeutic agents for inflammation.

**Supplementary Information:**

The online version contains supplementary material available at 10.1038/s41598-025-17841-1.

## Introduction

Heterocyclic chemistry has historically been the most important branch of organic chemistry. Moreover, it is the existing applied branch of organic chemistry that has played a vital role in a society from a pharmacological and industrial perspective, resulting in a majority of scientific research being devoted to this field^1–4^. Among the various kingdoms of nature, heterocyclic compounds are the most abundant; they account for more than half of all identified organic analogues. Heterocyclic scaffolds with N and S atoms are responsible for their unique biological properties, which are mostly utilized in the pharmaceutical industry. Chemically modified heterocyclic compounds are becoming more efficient in several biological aspects. It has stimulated us to enhance our quality of life by comprehending living organisms at the molecular level. These are found in a wide range of natural resources and are involved in various reactions^5^. They can also act as acids or bases based on certain media due to the presence of heteroatoms. Moreover, a few of them can be approached by electrophilic reagents, while others can act as nucleophilic reagents. Some heterocycles will be quickly oxidized and resistant to reduction, which will be contrary to others. These features influence the biologically active heterocyclic molecules, which depend on the heterocyclic system. The heteroatom is found to contain several factors, such as vitamins, antibiotics, hemoglobin, biologically important amino acids, proteins, nucleic bases, neurotransmitters, and a wide range of synthetic drugs and dyes containing heterocyclic rings^6^. The synthetically modified molecule has a pyridine heterocyclic ring system, and it holds a wide range of therapeutic roles, for example, analgesic, cardiovascular lipid management, anti-tuberculosis, anti-inflammatory activities, etc^7–9^.

For diabetes mellitus (DM), the main symptom is an increased blood sugar level. This is due to insulin resistance, relative insulin insufficiency, and enhanced endogenous glucose production (EGP)^10,11^. Controlling the rate at which glucose is released from food and preventing its absorption into the bloodstream is one possible technique for treating or preventing type 2 diabetes^12^. Elevated blood glucose levels characterize around 95% of diabetes cases due to insulin resistance^13^. The primary cause of prolonged insulin deficiency mortality in diabetic patients, which contributes to several complications, includes renal disease, neuropathy, retinopathy, and cardiovascular illnesses^14^. Prolonged insulin deficiency can cause several micro- and macrovascular cardiovascular dysfunctions to develop. A polygenic condition has similar physiological and pathological properties, including hypertension, heart disease, and diabetes, although type 1 and type 2 are different pathophysiological disorders^15^. Type 2 diabetes signifies cardiac dysfunction, and according to the WHO, there are approximately 422 million diabetic patients worldwide, and this number will increase by 700 million in 2045^16,17^.

Microbes, plants, and other organisms are all found to possess specific enzymes. α-Amylase is a metalloprotein that contains Ca^+ 2^ ions. In organisms, it is highly secreted in exocrine and pancreatic glands. α-Amylase breaks α-1,4-glucosidic linkages to promote the preliminary hydrolysis of carbohydrate complexes into mono- and disaccharide molecules. The brush border of the intestine contains the α-glucosidase enzyme, which is also extremely important. This produces free α-D-glucose, which is accountable for hyperglycemia. Nevertheless, two small internal organ membrane-bound enzymes, maltase-glucoamylase (MGA) and sucrose isomaltase (SI), are linked to α-glucosidase. These two are potent targets for inhibiting glucose in patients with type 2 diabetes because they are involved in the hydrolysis of dietary carbohydrates^18–20^. Elevating endogenous hypoglycemic agent secretion, improving insulin action in the target tissue, and lowering insulin demand. DM is treated by inhibiting the chemical conversion of oligo and disaccharides into monosaccharides^21,22^. Since α-amylase and α-glucosidase are vital for the digestion of carbohydrate complexes, inhibiting these enzymes may provide a desirable solution for managing postprandial hyperglycemia^23^. The commercially available α-amylase and α-glucosidase inhibitors are well established to treat type 2 diabetes mellitus disorder^24^. However, those clinical inhibitors exhibit substantial side effects, including stomach pain, flatulence, and liver disease for patients. As a result, it is important to discover α-amylase and α-glucosidase inhibitors with no or moderate adverse effects. Chronic activation of pro-inflammatory pathways in insulin action target cells may be a factor in obesity, insulin resistance, and associated metabolic diseases, such as type 2 diabetes. This is supported by accumulating data. A growing interest has been shown in addressing chronic inflammation to help prevent and control diabetes and related conditions, as well as to improve diabetes risk stratification by utilizing inflammatory biomarkers as potential indices, following the discovery of plausible pathways linking inflammation to diabetes.

Pyridine-based pharmacophores have an impact in medicinal chemistry due to their versatile biological activities. Recent studies highlight their promising enzyme inhibition potential. They exhibit significant inhibitory effects against key therapeutic targets such as kinases, aromatase, and α-glucosidase. These results underscore their potential in the development of treatments for cancer, metabolic disorders, and inflammatory diseases^25–27^. The present study aims to design and synthesize bioactive new nicotinic acid-based Pharmacophores **(2a-j)** examined for anti-inflammation and as an α-amylase inhibitor (anti-diabetic). From the outcome, the compound Benzo[d] [1,3] dioxol-5-yl nicotinate **(2 g)** derivative has a potential inhibitory effect on the α-amylase enzyme to introduce plausible therapeutic leads to serve as an anti-diabetic agent.

## Results and discussion

### Synthesis of phenyl nicotinate and nicotinamide derivatives

**Fig. 1:**
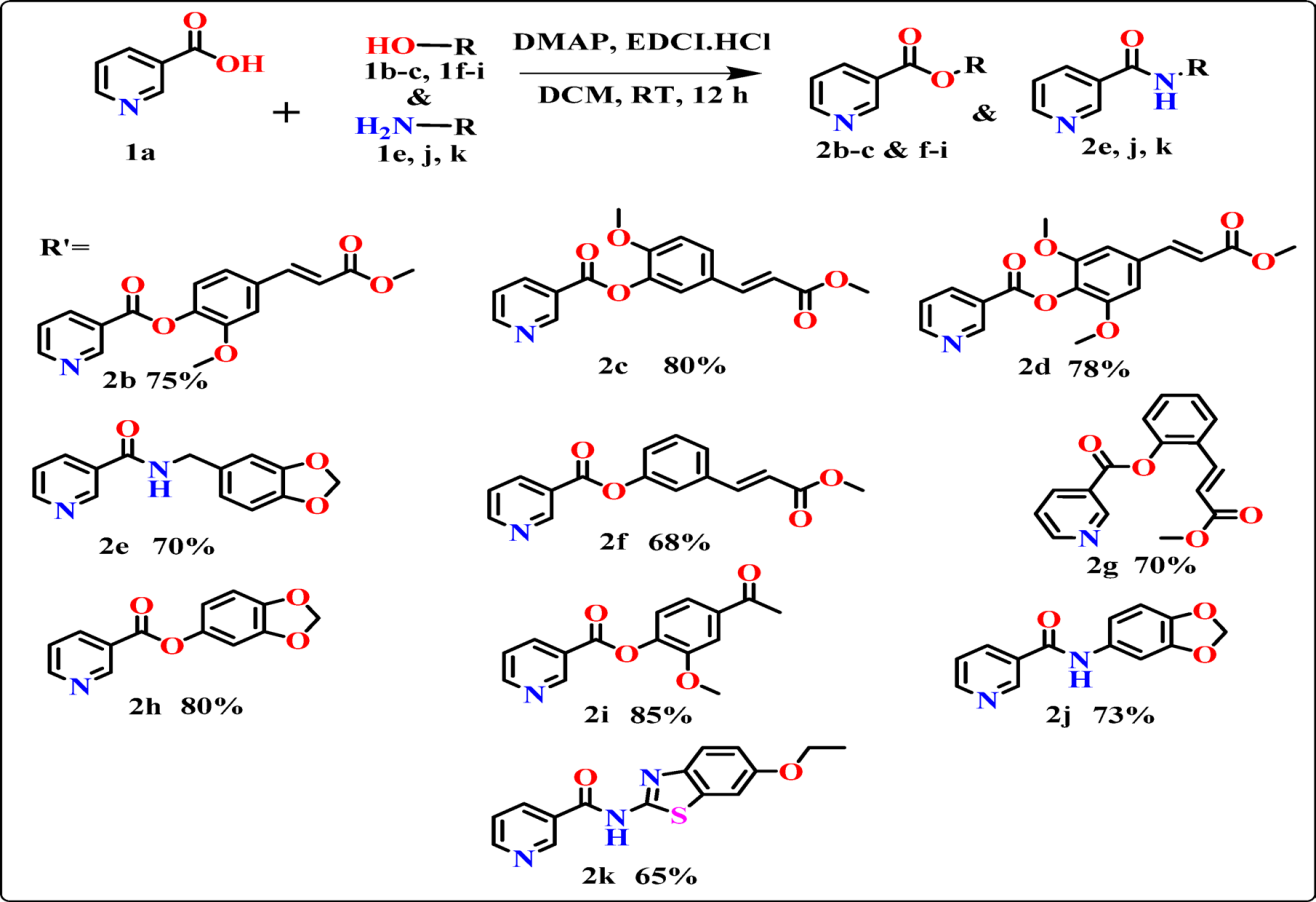
Synthesis of phenyl nicotinate derivatives (2b-k).

Substituted α, β-unsaturated phenol and amine derivatives were reacted with pyridine 3-carboxylic acid to produce corresponding phenyl nicotinate and nicotinamide derivatives (1–10) as displayed in Fig. 1. 4-dimethylaminopyridine acts as a nucleophilic base, and 1-(3-dimethylaminopropyl)-3-ethylcarbodiimide hydrochloride was used as a catalyst, and the reaction was monitored by TLC. Developed inhibitors are well-characterized by NMR, HRMS-ESI, and FTIR spectroscopic techniques. To the best of our knowledge, the structure of entities is found to be novel compounds.

### Study of single crystal X-ray diffraction

**Fig. 2:**
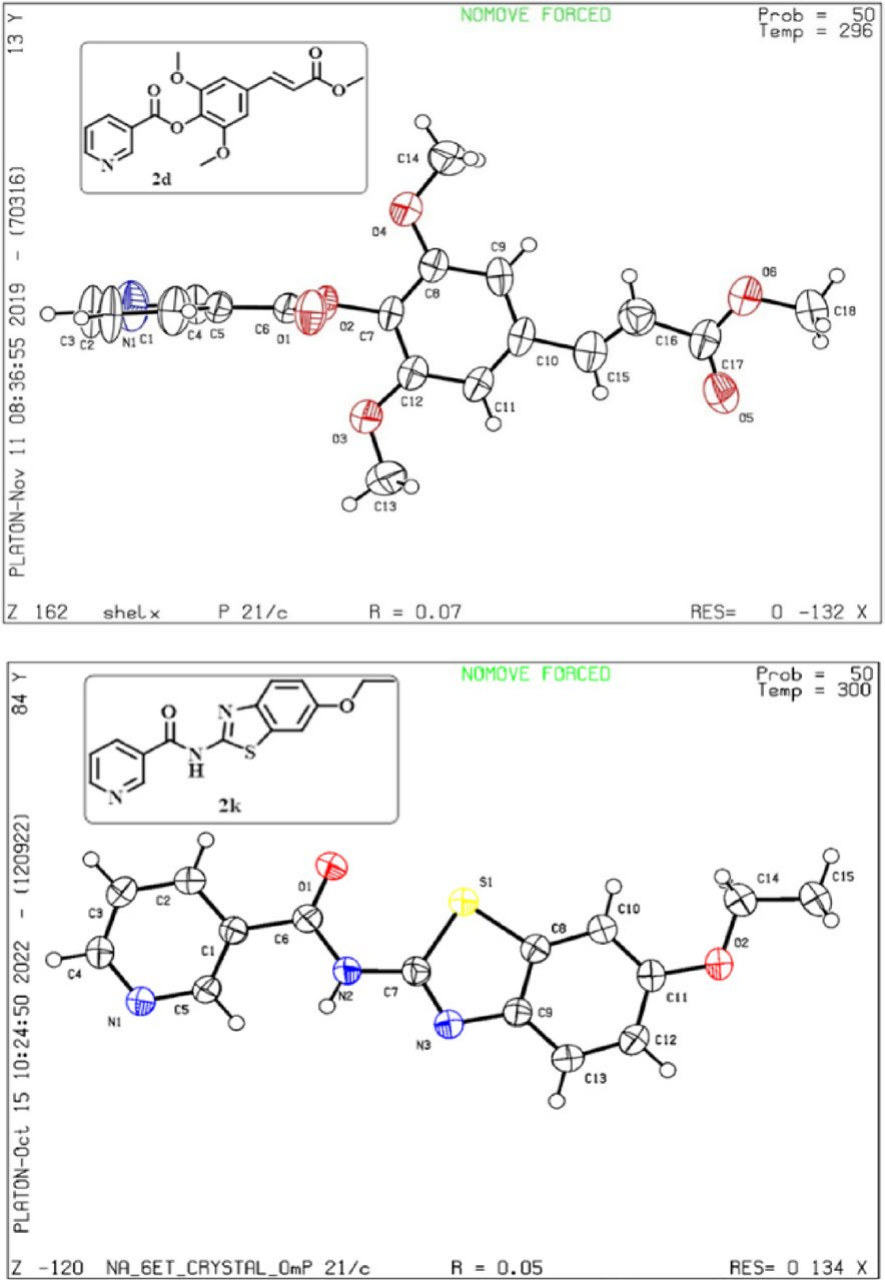
The ORTEP crystal image of compounds 2d and 2k refined by Olex2 structure solution software.

The synthesized compounds **(2d** and **2k)** are shown in Fig. 2 (ORTEP crystal image); the CCDC numbers are 1964977 **(2d)** and 2201942 **(2k).** The crystals of both compounds belong to the monoclinic structure. The space groups of the compounds 2d and 2k are P 2_1/C_ and P 1 21/c 1, respectively. The empirical formula is C_19_H_18_O_6_N **(2d)**, C_15_H_13_N_3_O_2_S **(2k)**, Obtained mass: 343.34 (2d), 299.34 (2k). Designed a unit cell measurement a = 11.416(3) Å, b = 9.2671(17) Å, c = 16.580(3) Å and α = 90 º, β = 106.674(9) º, γ = 90 º (2d) and a = 7.0795(6) Å, α = 90°, b = 26.584(2) Å, β = 112.643(2) °, c = 8.0420(6) Å, γ = 90° (2k).

Crystal volume (CV) and calculated density were observed, CV-1680.3(6) Å^3^ and 1.353 Mg/m^3^ **(2c)**, 1396.9(2) Å^3^ and 1.423 g/cm^3^ **(2j)**, Z = 4, and absorption coefficient of 0.103 mm^- 1^ and 0.240 mm^- 1^. The range of data collection is 1.862º ≤ θ ≤ 28.256º and 2.85° ≤ θ ≤ 28.30°. A total of 13,185 and 39,301 reflections were collected, with 3956 unique single (R_int_ = 0.0364) and 3479 (R_(int)_ = 0.0651). Full-matrix least-squares made refinement methods on F2, and full-matrix least-squares enhanced non-hydrogen atoms to obtain a range of R_1_ = 0.1251 and R_2_ = 0.2436 (2d) and R_1_ = 0.0474, R_1_ = 0.0929 **(2k)**. The observed bond length, bonding angles, and other validating parameters are based on the single-crystal XRD data provided in Tables S1 and S4, respectively.

### In silico molecular modelling

**Fig. 3:**
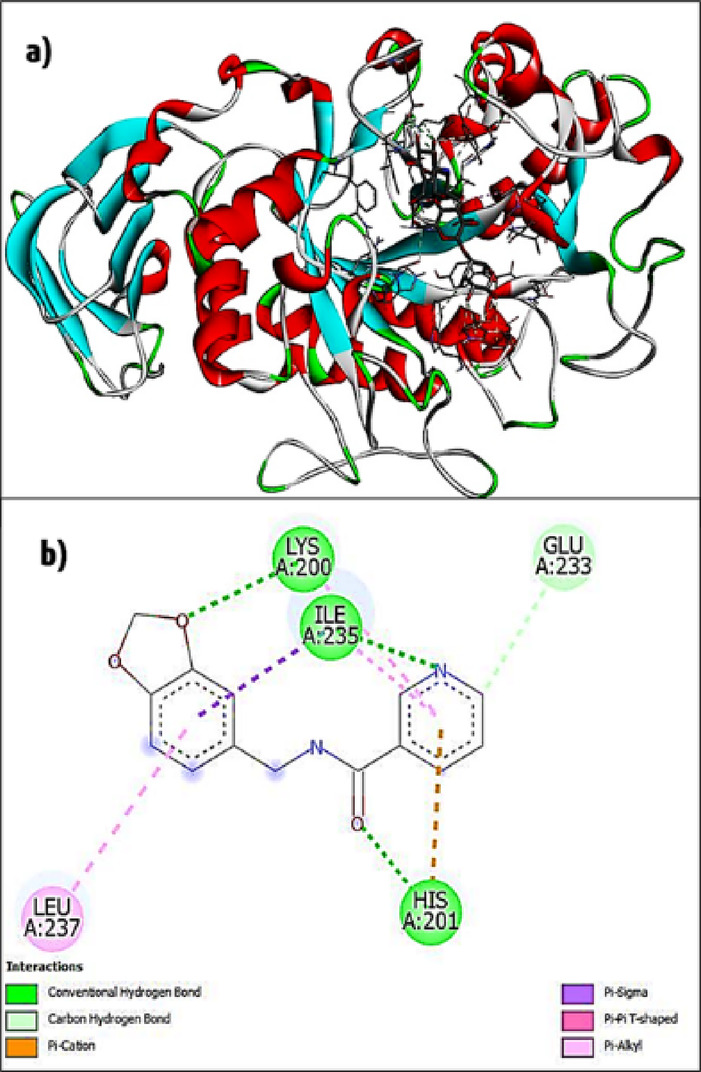
(**a**) 3D structure of α-amylase, (**b**) compound 2e. interactions were visualized by the Discovery Studio R2 software.

The molecular docking results of the designed molecules exposed that all new analogs exhibited different types of intermolecular binding with the target enzymes of the active site amino acid residues. The binding interactions and energies of the synthesized inhibitors and α-amylase enzyme complex are given in Table 1. In the first, we re-docked the commercially available, well-known drug (acarbose) in the binding site of α-amylase with the same parameters used for designed inhibitors. The acarbose drug showed eight bonding interactions with the amino-acid residues Trp A:58, A:59, Try A:151, Leu A:162, Lys A:200, Glu A:233, Glu A:240, and Asp 300 of the enzyme (Fig. 3). The structure of the acarbose-α-amylase complex binding interaction value is -6.66 kcal/mol. Further, the synthesized inhibitors were docked with α-amylase; this study carried out the same procedure we used for prediction in the positive control. The results revealed binding interactions between inhibitor molecules and the enzyme-active site of the amino acids. From the docking results, the lowest binding interaction energies of α-amylase (5.55 and 5.60 kcal/mol^-1^) were observed after the docking study. Among the 10 derivatives, three compounds **2e**, **2f**, and **2k**, showed the highest binding interaction scores (-6.86, -6.50, and − 7.18, respectively). The designed molecules **2e**, **2f**, and **2k** docked into the corresponding enzyme target reveal that several inhibitory molecular interactions were assumed to be accountable for the significant affinity of these docked molecules. Molecule **2k** is the most active complex formation, and it is the respective target. It has interacted with active site amino acids to form two conventional hydrogen bonds, such as Arg A:195 and Lys A:200, and carbon-hydrogen bond (Glu A:233) interactions.

The electronegative carbonyl group of 6-ethoxy benzothiazole and pyridine rings forms π-cation, π-anion (Asp A:197, His A:201, Glu A:233, and Asp A:300), and π-sulfur (His A:201), as well as other forms such as π-π-T-shaped (Tyr A:151, Leu A:162) and alkyl, π-alkyl (Ala A:198, Lys A:200, and Ile A:235) interactions. The compounds **2e** and **2f** exhibited similar binding energies (-6.86 and − 6.50 kcal/mole^-1^) compared with the positive control (acarbose). Moreover, the active amino acids interact with synthesized inhibitor molecules and form an enzyme-receptor complex. These compounds display three conventional hydrogen bonds, such as Lys A:200, His A:201, and Ile A:235, and a carbon-hydrogen bonding interaction Glu A:233, π-cation His A:201, π-sigma Ile A:235, π-π-T-shaped Leu A:237, and π-alkyl Ile A:235 and Lys A:200 interactions (molecule **2e**) (Figs. 3 & S45).

**Table 1:** Binding affinity of α-amylase enzyme with designed inhibitors 2b-k.

Ligands	∆G (Kcal/mol)	Key interactions
Acarbose	-6.66	5- Conventional hydrogen bonds, 4- carbon-hydrogen bonds, 1- alkyl
2b	-5.60	2- Conventional hydrogen bonds, 2- carbon-hydrogen bonds, 1- Pi-Sigma, 1- Pi-Cation
2c	-6.28	1- Conventional hydrogen bond, 2- carbon-hydrogen bonds, 2- Pi-Sigma, 3- Alkyl
2d	-5.72	2- Conventional hydrogen bonds, 1- Pi-Sigma, 1- Pi-Anion, 2- Pi-Alkyl
2e	-6.86	3- Conventional hydrogen bonds, 1- carbon-hydrogen bond, 1- Pi-Cation, 1- Pi-Alkyl
2f	-6.50	1- Conventional hydrogen bond, 1- carbon-hydrogen bond, 2- Pi-Anion, 3-Alkyl
2 g	-6.02	3- carbon-hydrogen bonds, 2- Pi-Anion, 1- Alkyl, 1- Pi-Pi Stacked
2 h	-5.55	3- Conventional hydrogen bonds, 1- carbon-hydrogen bond, 1- Pi-Pi stacked
2i	-5.60	2- Conventional hydrogen bonds, 1- carbon-hydrogen bond, 3- Pi-Alkyl, 1- Pi-Sigma, 1- Pi-Cation
2j	-6.09	3- Conventional hydrogen bonds, 1- carbon-hydrogen bond, 1- Pi-Sigma, 2- Pi-Cation, 1- Pi-Pi T-shaped
2k	-7.18	2- Conventional hydrogen bonds, 1- carbon-hydrogen bond, 3- Pi-Sulfur, 4- Alkyl

### In vitro Inhibition study against α-amylase enzyme

The characterized inhibitors **2b-k** exposed well in vitro inhibition against the α-amylase receptor, having a half-maximal inhibitory concentration efficacy (IC_50_) range of 1.324 ± 0.17–1.516 ± 0.14 µg as compared to the drug acarbose (IC_50_ = 1.273 ± 0.12 µg), respectively. Commonly, the synthesized ligands were comprised of nicotinate and nicotinamide bonds. Moreover, the different substituted ester and amine derivatives were vital to the potential inhibitor.

#### Structure-activity relationship (SAR) between the inhibitors and α-amylase enzyme

The structural properties of all evaluated inhibitor components, such as the niacin moiety (pyridine ring), nicotinamide, phenyl nicotinate, and different substituted phenol and amine derivatives, play significant roles in the α-amylase inhibition study. However, the substituted phenolic and amine derivatives exhibited variable results while testing for target enzyme inhibition activity (Fig. S41). A limited SAR was elucidated based on the different substituted chemical variables and their attachment to positions.

The commercial acarbose drug is used as a positive control to inhibit activity, resulting in IC_50_ = 1.273 ± 0.12 µg. All synthesized molecules have shown good inhibitory activity against the α-amylase enzyme (Table S2). Among them, compounds **2e** (IC_50_ = 1.324 ± 0.21 µg) and **2j** (IC_50_ = 1.326 ± 0.10 µg) exhibited more inhibitory activity compared to other derivatives because of a substituted amide bond and methylenedioxyphenyl, and this function contains many pharmacological features for preventing cardiovascular-related disorders.

Compound **2d** (IC_50_ = 1.346 ± 0.24 µg) with sinapic ester substitution was less active than compounds **2e** and **2j**. Meta-coumaric ester (**2f**, IC_50_ = 1.334 ± 0.17 µg) substitution showed similar inhibitory activity, which was observed when compared with **2e** and **2j**. Inhibitor molecule ferulic ester substitution **2b** (IC_50_ = 1.368 ± 0.12 µg) exhibited decreased α-amylase activity, and **2 g** (IC_50_ = 1.357 ± 0.15 µg) ortho-substituted coumaric ester derivative also showed similar activity. Compared to active inhibitors (**2e** and **2j**), the following other substituted inhibitor molecules, such as **2c** (IC_50_ = 1.405 ± 0.25 µg), **2 h** (IC_50_ = 1.516 ± 0.14 µg), **2i** (IC_50_ = 1.423 ± 0.24 µg), and **2k** (IC_50_ = 1.515 ± 0.17 µg), were found to be less potent against the α-amylase. These inhibitory active molecules (**2b**-**k**) exhibited comparable activity with each other against the α-amylase protein, but slight variation in inhibition results was observed due to the presence of different substitutions of the inhibitors (Table S3); nevertheless, the least active molecule has the lower possibilities of interaction with the active site of the amino acids.

### SAR of antiradical activity profiling

The antioxidant inhibition efficacy of the synthesized inhibitor molecules by the ABTS and DPPH methods is provided in Table S2. The experimental results were reported in half-maximal inhibition efficiency (IC_50_) values and positive control as ascorbic acid (IC_50_ = 11.81 ± 0.04 µM, DPPH, and 11.90 ± 0.01 µM, ABTS) for DPPH (IC_50_ = 12.88 ± 0.19 to 99.62 ± 0.15 µM) and ABTS (IC_50_ = 16.35 ± 0.25 and 88.03 ± 0.17 µM) methods (Table S2). The synthesized compounds exhibited moderate inhibitory activity in both cases, DPPH and ABTS (Figs. S42 and S43). Among all derivatives, compounds **2b** and **2 h** showed excellent inhibition efficiency with IC_50_ values of 15.63 ± 0.13 µM and 12.88 ± 0.19 µM (DPPH) IC_50_ = 19.89 ± 0.25 µM and 16.35 ± 0.25 µM (ABTS), which is in good agreement with the standard ascorbic acid as a positive control (11.81 ± 0.04 µM (DPPH) and 11.90 ± 0.01 µM (ABTS)). Compound **2d** (IC_50_ = 32.02 ± 0.26 µM) displayed moderate activity against DPPH, and all synthesized derivatives exhibited the least activity compared with standard drugs. In ABTS analysis, the inhibitory compounds **2c** (IC_50_ = 20.57 ± 0.13 µM), **2d** (IC_50_ = 21.57 ± 0.11 µM), **2 g** (IC_50_ = 21.85 ± 0.21 µM), and **2i** (IC_50_ = 38.43 ± 0.21 µM) showed good activity against ABTS cationic radicals, and other inhibitor molecules **2f** and **2k** displayed the lowest inhibition efficiency while compared with the positive control (Table S3). The anticipation of radical scavenging assay was performed to evaluate the tendency of anti-oxidant behavior and reproducible results for the synthesized inhibitor molecules.

The experimental method principle relies on the degradation of the purple color of the DPPH radical and the blue color reduction of the ABTS cation radical. This is due to consuming the electron or hydrogen radical from the antioxidant (A-H). Both assays were measured at 517 nm and 734 nm, respectively. The distinction of color changes illustrates the potential scavenging activity of the inhibitor molecules in terms of hydrogen-donating proficiency. From this, we can conclude the anti-oxidant efficacy.

### SAR of ex vivo anti-inflammatory activity

The synthesized inhibitors were exposed to anti-inflammatory activity by induced human blood sample (hypotonic solution-induced hemolysis of erythrocyte membrane), and the results are displayed in Fig. S44. Based on the inhibition efficiency (Table S3), the IC_50_ values were evaluated and compared with the standard drug (ketorolac). Table S2 illustrates the evaluated IC_50_ values (IC_50_ = 14.06 ± 0.15 to 85.56 ± 0.25 µM) of all synthesized inhibitor molecules with positive control as ketorolac (IC_50_ = 11.79 ± 0.17 µM). The anti-inflammatory properties of the developed inhibitors were revealed based on the concentrations because the observation of anti-inflammatory activity results shows the concentration was increased, and the inhibition efficiency also increased. Amongst the inhibitors **2b** (IC_50_ = 18.41 ± 0.13 µM) and **2e** (IC_50_ = 14.06 ± 0.15 µM) exhibited excellent inhibition against the induced human RBC hemolysis sample. Compound **2e** (IC_50_ = 18.41 ± 0.13 µM) showed good activity due to the amide bond and methylenedioxy groups, which exhibit comparable activity with the positive control (IC_50_ = 11.79 ± 0.17 µM) as ketorolac.

Other substitution inhibitors such as **2c** (IC_50_ = 27.65 ± 0.20 µM), **2d** (IC_50_ = 20.23 ± 0.26 µM), **2 h** (IC_50_ = 21.69 ± 0.24 µM), **2j** (IC_50_ = 20.28 ± 0.22 µM) and **2k** (IC_50_ = 25.86 ± 0.23 µM) showed similar RBC hemolysis activity, among this compound **2d** has methoxy substitutions, and it may involve a similar activity, and compound **2j** has amide and methylenedioxy functions are present so it might be involved and decreased the inflammatory activity. Compared to other derivatives, the compounds **2 g** (IC_50_ = 85.56 ± 0.25 µM) and **2i** (IC_50_ = 66.97 ± 0.28 µM) exhibited the least inhibition efficiency of RBC hemolysis.

The results clearly indicate that the novel nicotinic acid derivatives reported in this study may be useful in treating diabetes-related complications. Further elaborate study with in vivo model with specific assays may throw some light on the further therapeutic development against diabetes.

### Toxicity evaluation

The toxicity of the molecule was investigated by the MTT cell viability assay employing RAW macrophage cells. For this study, two lead compounds, **2b** and **2e**, were screened based on the in vitro results. The outcome of the assay indicates that the compound is viable for the cell line. (The OD values in Tables S6 and S7)

### Limitations

The nicotinic acid derivatives, though biologically active, exhibited certain limitations when compared to standard drugs. Firstly, their inhibitory activity against α-amylase and inflammatory mediators was lower, indicating reduced potency. The pharmacokinetic properties, such as solubility and bioavailability, may also be suboptimal, which can affect their therapeutic efficiency. Some derivatives showed moderate binding affinity in molecular docking, suggesting weaker interaction with target proteins. Additionally, their selectivity profile was not as robust as that of commercial drugs, raising concerns about off-target effects. Cytotoxicity was observed at higher concentrations in a few analogs, limiting their therapeutic window.

## Experimental

### Materials and methods

Starting chemicals were purchased through respected dealers and used without additional purification. The^1^H NMR and ^13^C NMR spectra were taken on a Bruker NMR 400 MHz spectrometer using the deuterated solvents (CDCl_3_, DMSO‑d_6_). FT-IR spectra were derived from Shimadzu IR-affinity-1 with a resolution IV spectrometer for samples in KBr discs. The RAW 264.7 cell line was supplied by American Type Culture Collection (ATCC), and the cell culture medium was purchased from Merck. On the WATERS-XEVO G2-XS-QT (HRMS spectra), the molecular mass of the inhibitors was validated using a Perkin Elmer GC Clarus 680- MS Clarus 600 Turbo Mass Ver 5.4.2 (GC-MS) (Figs. S1–S40). The JASCO UV-Vis-NIR V-670 spectrophotometer was used to acquire UV-visible spectral characteristics at 540 nm (amylase), 560 nm (inflammatory), 517 nm (DPPH), and 734 nm (ABTS). The crystal structure of the compound was analyzed in Bruker Kappa Apex II Model, X-Shell with Olex-2 structure solution software (**2c**). Crystal data with details on data collection and structure refinement are provided in Table S4.

### General procedure

A mixture of aromatic acid (1 mmol) and aromatic alcohol (2 mmol) was taken in a 50 mL RB flask, followed by 4-dimethylaminopyridine and 1-(3-dimethylaminopropyl)-3-ethylcarbodiimide hydrochloride (2.2 mmol), and 5 mL of DCM was added to the reaction mixture. The mixture was stirred at room temperature for 12 h. The reaction was monitored by TLC, and after completion of the reaction, the mixture was extracted with ethyl acetate/water. Then the separated organic layer was dried over Na_2_SO_4_. The mixture was purified by column chromatography, the eluent was a mixture of hexane/ethyl acetate (8:2).

#### 2-Methoxy-4-(3-methoxy-3-oxoprop-1-en-1-yl) phenyl nicotinate (2b)

 Yield – 75%, mp: 120 ^◦^C, FT-IR: C-H = 2941.44, C = O = 1735.93, C = C = 1635.64, Ar-C-*N* = 1325.10, C-O = 1211.30^1^. H NMR (400 MHz, CDCl_3_) δ 9.40 (s, 1 H), 8.70–8.85 (dd, 1 H), 8.46 (d, *J* = 7.9 Hz, 1 H), 7.71–7.67 (d, *J* = 16.0 Hz, 1 H), 7.49–7.46 (dd, *J* = 7.9 Hz, 2 H), 7.19–7.16 (d, *J* = 12.2 Hz, 3 H), 6.45–6.41 (d, *J* = 16.0 Hz, 1 H), 3.86–3.82 (d, 6 H)^13^. C NMR (100 MHz, CDCl_3_) δ 167.25, 163.24, 154.02, 151.52, 151.44, 144.07, 141.14, 137.79, 133.74, 125.24, 123.50, 123.26, 121.23, 118.29, 111.38, 55.97, 51.83. Calculated mass (*m/z*): 313.0950, Obtained mass: 313.1000.

#### 2-Methoxy-5-(3-methoxy-3-oxoprop-1-en-1-yl) phenyl nicotinate (2c)

Yield – 80%, mp: 156–158 ^◦^C, FT-RI: C-H = 2941.44, C = O = 1735.93, C = C = 1635.64, Ar-C-*N* = 1325.10, C-O = 1211.30^1^.  H NMR (400 MHz, CDCl_3_) δ = 9.40 (s, 1 H), 8.87–8.85 (dd, 1 H), 8.47–8.45 (d, *J* = 7.9 Hz, 1 H), 7.66–7.62 (d, *J* = 16.0 Hz, 4 H), 7.49–7.42 (m, 2 H), 7.38 (s, 1 H), 7.03–7.01 (d, *J* = 8.5 Hz, 4 H), 6.34–6.30 (d, *J* = 16.0 Hz, 4 H), 3.86 (s, 3 H), 3.79 (s, 3 H)^13^. C NMR (100 MHz, CDCl_3_): δ = 167.47, 163.28, 154.05, 152.87, 151.52, 143.55, 139.78, 137.76, 128.05, 127.64, 125.21, 123.50, 121.92, 116.59, 112.48, 56.09, 51.71. Calculated mass (*m/z*): 313.0950, Obtained mass: 313.1061.

#### 6-Dimethoxy-4-(3-methoxy-3-oxoprop-1-en-1-yl) phenyl nicotinate (2d)

 Yield – 78%, mp: 160 ^◦^C, FT-RI: C-H = 3014-74, C = O = 1735.93, C = C = 1635.64, Ar-C-*N* = 1278.81, C-O = 1253.73^1^.  H NMR (400 MHz, CDCl_3_) δ = 9.41 (s, 1 H), 8.85–8.84 (t, 1 H), 8.48–8.46 (d, *J* = 7.9 Hz, 1 H), 7.67–7.63 (d, *J* = 15.9 Hz, 1 H), 7.48–7.45 (d *J* = 7.4 Hz, 1 H), 6.82 (s, 2 H), 6.44–6.40 (d, *J* = 15.9 Hz, 1 H), 3.84–382 (s, 9 H)^13^. C NMR (100 MHz, CDCl_3_) δ = 167.19, 163.02, 153.90, 152.52, 151.61, 144.51, 137.86, 133.07, 130.02, 125.24, 123.44, 118.31, 104.65, 56.22, 51.82. Calculated mass (*m/z*): 343.1056, Obtained mass: 343.1100.

#### N-(Benzo[1,3]dioxol-5-ylmethyl)nicotinamide (2e)

Yield – 70%, mp: 96–98 ^◦^C, FT-RI: NH = 3309.85, C-H = 2914.44, C = O = 1662.64, C = C = 1595.13, Ar-C-*N* = 1273.02, C-O = 1242-16^1^.  H NMR (400 MHz, CDCl_3_) δ = 8.98 (d, 1 H), 8.70 (s, 1 H), 8.14 (d, *J* = 7.9 Hz, 1 H), 7.38–7.37 (d, 1 H), 6.76 (s, 1 H) 6.73 (d, 2 H), 5.94 (d, 2 H), 4.31 (d, 2 H)^13^. C NMR (100 MHz, CDCl_3_) δ = 169.92, 152.27, 147.92, 146.96, 135.22, 132.10, 123.50, 121.13, 108.45, 108.30, 101.15, 101.09, 44.02, 43.57. Calculated mass (M + 1): 256.0848, Obtained mass: 257.0951.

#### 3-(3-Methoxy-3-oxoprop-1-en-1-yl)-phenyl nicotinate (2f)

Yield – 68%, mp: 98 ^◦^C, FT-IR: C-H = 2949.16, C = O = 1739.79, C = C = 1633.71, Ar-C-*N* = 1259.52, C-O = 1217.08^1^.  H NMR (400 MHz, CDCl_3_) δ = 9.40 (d, 1 H), 8.88–8.86 (d, 1 H), 8.47- 8,45 (d, *J* = 8.0 Hz, 1 H), 7.71–7.67 (d, *J* = 16.0 Hz, 2 H), 7.51–7.40 (m, 3 H), 7.40 (s, 2 H), 7.28–7.25 (q, *J* = 6.9 Hz, 1 H), 6.48–6.44 (d, *J* = 16.0 Hz, 2 H), 3.81 (s, 3 H)^13^. C NMR (100 MHz, CDCl_3_) δ = 167.11, 163.76, 154.19, 151.40, 150.90, 143.57, 137.67, 136.22, 130.11, 126.09, 125.35, 123.56, 123.38, 120.80, 119.19, 51.86. Calculated mass (*m/z*): 283.0845, Obtained mass: 283.0990.

####  2-(3-Methoxy-3-oxoprop-1-en-1-yl)phenyl nicotinate (2 g)

Yield – 70%, mp: 96 ^◦^C, FT-RI: C-H = 2951.09, C = O = 1735.93, C = C = 1639.49, Ar-C-*N* = 1269.16, C-O = 1217.08^1^. H NMR (400 MHz, CDCl_3_) δ = 9.43 (d, 1 H), 8.89–8.87 (dd, *J* = 8.0 Hz, 1 H), 8.49- 8,47 (tt, *J* = 8.0 Hz, 1 H), 7.81–7.77 (d, *J* = 16.0 Hz, 1 H), 7.72–7.69 (d, *J* = 12.0 Hz, 1 H), 7.52–7.45 (m, 2 H), 7.36–7.32 (t, 1 H), 7.26–7.24 (d, *J* = 8.0 Hz, 2 H), 6.50–6.46 (d, *J* = 16.0 Hz, 2 H), 3.83 (s, 3 H)^13^. C NMR (100 MHz, CDCl_3_) δ = 167.95, 163.67, 154.34, 151.48, 149.05, 137.75, 131.31, 127.78, 127.37, 126.81, 125.03, 123.67, 123.09, 120.37, 51.83. Calculated mass (*m/z*): 283.0845, Obtained mass: 283.1000.

#### Benzo[1,3]dioxol-5-yl nicotinate (2 h)

Yield – 80%, mp: 92 ^◦^C, FT-RI: C-H = 2891.30, C = O = 1724.36, C = C = 1589.34, Ar-C-*N* = 1284.59, C-O = 1242-16^1^. H NMR (400 MHz, CDCl_3_) δ = 9.37 (d, 1 H), 8.86–8.84 (dd, *J* = 8 Hz, 1 H), 8.44–8.42 (tt, *J* = 8.0 Hz, 1 H), 7.48–7.45 (m, *J* = 7.9 Hz, 1 H), 6.84 (d, *J* = 8.4 Hz, 1 H), 6.74 (d, 1 H), 6.68–6.65 (dd, *J* = 8.4 Hz 1 H), 6.02 (s, 2 H)^13^. C NMR (100 MHz, CDCl_3_) δ = 164.19, 154.01, 151.35, 148.18, 145.71, 144.74, 137.62, 125.52, 123.49, 113.97, 108.12, 103.71, 101.86. Calculated mass (M + 1): 243.0532, Obtained mass: 244.0635.

#### 4-Acetyl-2-methoxyphenyl nicotinate (2i)

Yield – 85%, mp: 110 ^◦^C, FT-RI: C-H = 2943.37, C = O = 1734.01, C = C = 1672.28, Ar-C-*N* = 1278.81, C-O = 1234.44^1^.  H NMR (400 MHz, CDCl_3_) δ = 9.41 (s, 1 H), 8.87–8.86 (d, 1 H), 8.47–8.45 (d, *J* = 7.9 Hz, 1 H), 7.66–7.61 (t, 2 H), 7.50–7.47 (dd, *J* = 7.8 Hz, 1 H), 7.28–7.26 (t, 1 H), 3.89 (s, 3 H), 2.63 (s, 3 H)^13^. C NMR (100 MHz, CDCl_3_) δ = 196.96, 163.01, 154.13, 151.55, 151.45, 143.53, 137.81, 136.27, 125.10, 123.53, 122.82, 122.01, 111.55, 56.11, 26.62. Calculated mass (*m/z*): 271.0845, Obtained mass: 271.0800.

#### N-(Benzo[1,3]dioxol-5-yl)nicotinamide (2j)

Yield – 73%, mp:190–192 ^◦^C^1^,  H NMR (400 MHz, CDCl_3_) δ = 9.80 (s, 1 H), 9. 18 (s, 1 H), 8.73 (d, 1 H), 8.28–8.26 (d, 1 H), 7.43–7.39 (m, 2 H), 7.15 (d, *J* = 8.3 Hz, 1 H), 6.78 (d, *J* = 8.4 Hz, 1 H), 5.97 (s, 2 H)^13^. C NMR (100 MHz, CDCl_3_) δ = 164.18, 151.88, 148.85, 147.47, 144.13, 135.55, 132.76, 130.95, 123.18, 113.91, 107.84, 103.27, 101.07. Calculated mass (*m/z*): 242.0691, Obtained mass: 242.1404.

#### N-(6-ethoxybenzothiazol-2-yl) nicotinamide (2k)

Yield – 65%, mp: 240 ^◦^C, FT-RI: N-H = 3381.21, C-H = 2951.09, C = O = 1737.86, C = C = 1693.50, Ar-C-*N* = 1288.45, C-O = 1259.52, N = C-S = 1213.23^1^.  H NMR (400 MHz, DMSO) δ = 13.00 (s, 1 H), 9.24 (d, 1 H), 8.81–8.80 (d, 1 H), 8.46–8.44 (d, *J* = 8.0 Hz, 1 H), 7.69–7.67 (d, *J* = 8.8 Hz, 1 H), 7.62 (m, *J* = 7.4 Hz, 2 H), 7.07–7.05 (dd, *J* = 8.8 Hz, 1 H), 4.12–4.07 (q, *J* = 7.0 Hz, 2 H), 1.37–1.34 (s, 3 H)^13^. C NMR (100 MHz, DMSO) δ = 165.12, 156.07, 153.48, 149.71, 136.44, 133.20, 128.59, 124.09, 116.00, 105.86, 64.12, 15.16. Calculated mass (M + 1): 299.0728, Obtained mass: 300.0833.

### Computational and biological investigation of synthesized entities (2b-k)

#### In silico docking methodology

The Auto Dock Tools (ADT) software was used to dock all of the designed inhibitors against the α-amylase target by using the molecular docking model strategy (Table S5). The potential of ligand interactions between the designed ligand and α-amylase was investigated by molecular docking. The Protein Data Bank (http://www.rcsb.org/pdb) was used to obtain the crystalline structures of α-amylase (PDB code: 5KEZ).

The protein structure was prepared by removing water molecules from the crystal structure of 5KEZ in order to establish the potential nicotinic acid receptor. The 3D structure of niacin derivatives was developed by Chem 3D 20.1.1 software. Discovery Studio R2 software was used to explore the interaction modes, and docking results were used to evaluate the factors (such as binding energy and hydrogen bond distances).

#### In vitro inhibitory action of DPPH and ABTS radicals

Methanolic DPPH solution (0.15 mM) was prepared, and 1.0 mL of DPPH solution was mixed with 3.0 mL of synthesized molecules at different concentrations (20 µM to 100 µM). The mixture was constantly swirled and stored RT for 30 min, followed by absorbance measurement at 517 nm.

In brief, 7.0 mM ABTS in H_2_O and 2.5 mM potassium persulfate (1:1) were shaken well to generate ABTS cation radicals and kept in the dark at RT for 15–20 h before it is used for the experiment. After, the radical was mixed with the synthesized molecules at different concentrations (20 µM to 100 µM). The resultant solution was measured for absorbance at 734 nm. Ascorbic acid is the positive control for this experiment.

1 $$\:\varvec{\%}\:\varvec{i}\varvec{n}\varvec{h}\varvec{i}\varvec{b}\varvec{i}\varvec{t}\varvec{i}\varvec{o}\varvec{n}=\:\frac{\varvec{c}\varvec{o}\varvec{n}\varvec{t}\varvec{r}\varvec{o}\varvec{l}-\varvec{t}\varvec{e}\varvec{s}\varvec{t}\:\varvec{s}\varvec{a}\varvec{m}\varvec{p}\varvec{l}\varvec{e}}{\varvec{c}\varvec{o}\varvec{n}\varvec{t}\varvec{r}\varvec{o}\varvec{l}}\:\times\:100$$

#### Enzyme inhibition study

α-Amylase enzyme and other chemical reagents are used for enzyme inhibitory activity. The characterized compounds are diluted in an analytical grad solvent (DMSO) system at 1 mg/mL solution concentration. The activity reaction of the synthesized analogues on the enzyme was treated at final concentrations of 2 to 10 µg. In the interaction between enzyme and compounds, 10 min incubation was maintained before the inhibitory activity started. The enzyme-receptor inhibition efficiency was calculated and compared with the positive control. The activity responses plotted inhibition % Vs compounds concentrations (2–10 µg) and the IC_50_ values were evaluated from obtained inhibition activity results.

##### α-amylase inhibitory action

The design and synthesized analogues were evaluated against the EC.3.2.1.1 (α-amylase) enzyme adopted by the previously described method^28^ and compared with acarbose as a standard drug molecule. Sodium phosphate buffer 0.2 mM (pH 6.9 with 6 × 10^− 4^ M NaCl) in 500 µL enzyme solution (0.5 mg/mL) was taken and incubated with test samples (2–10 µg/mL) at room temperature for 10 min. A starch (1%) solution of 500 µL with phosphate buffer was added to all test inhibitor samples; further, the tubes were incubated for 10 min at 28 °C. The reaction test tubes were incubated for another 5 min in the water bath (100 °C) after the addition of the color reagent dinitro salicylic acid and cooled at room temperature until reached. Finally, 10 mL of distilled water was mixed with each test tube, and the colored mixture was measured at a fixed wavelength of 540 nm. The absorbance without sample solution (blank) and control (buffer and standard drug sample) was measured at 540 nm. The α-amylase inhibition efficiency was validated using the following equation:

2 $$\:\varvec{\%}\:\varvec{i}\varvec{n}\varvec{h}\varvec{i}\varvec{b}\varvec{i}\varvec{t}\varvec{i}\varvec{o}\varvec{n}=\:\frac{\varvec{c}\varvec{o}\varvec{n}\varvec{t}\varvec{r}\varvec{o}\varvec{l}-\varvec{t}\varvec{e}\varvec{s}\varvec{t}\:\varvec{s}\varvec{a}\varvec{m}\varvec{p}\varvec{l}\varvec{e}}{\varvec{c}\varvec{o}\varvec{n}\varvec{t}\varvec{r}\varvec{o}\varvec{l}}\:\times\:100$$

#### Anti-inflammatory activity

All synthesized compounds are validated in human RBC (blood sample); the blood sample was collected from a healthy volunteer in an EDTA tube. The EDTA tube further proceeded with centrifugation at 3000 rpm for 30 min. A supernatant (which contains plasma and leucocytes) solution was isolated, and the obtained RBC (red blood cells) was further treated with Alsever’s reagent. Alsever’s reagent was prepared as follows: sodium citrate (0.8 g, 2.6 mmol), dextrose (2 g, 11.0 mmol), citric acid (0.050 g, 0.02 mmol), and sodium chloride (0.42 g, 7.0 mmol) were solubilized in 100 mL of distilled water. The above mixture was centrifuged for 30 min at 3000 rpm. Then, colloidal blood cells were separated, carefully transferred, and washed with 0.85% of the isosaline solution; this process continued until a clear solution appeared. Employing coagulated RBC and generated human erythrocyte hemolysis, the anti-inflammatory activity and potential of the synthesized entities were evaluated. The tested compounds are prepared at different concentrations for the inhibition activity of inflammation, along with 100 µL of a purified RBC sample utilized. All characterized samples were followed by adding 5.0 mL of trisodium phosphate (10 mM) and 5.0 mL of sodium chloride (5.0 mM) buffer solutions. After the addition of the above mixtures, the test samples are incubated for 15 min at room temperature with RBC and hypotonic mixtures. After incubation, the mixture was centrifuged at 3000 rpm for 15 min. The final supernatant liquid solution was measured at 560 nm using a UV-visible spectrophotometer.

3 $$\:\varvec{\%}\:\varvec{i}\varvec{n}\varvec{h}\varvec{i}\varvec{b}\varvec{i}\varvec{t}\varvec{i}\varvec{o}\varvec{n}=\:\frac{\varvec{c}\varvec{o}\varvec{n}\varvec{t}\varvec{r}\varvec{o}\varvec{l}-\varvec{t}\varvec{e}\varvec{s}\varvec{t}\:\varvec{s}\varvec{a}\varvec{m}\varvec{p}\varvec{l}\varvec{e}}{\varvec{c}\varvec{o}\varvec{n}\varvec{t}\varvec{r}\varvec{o}\varvec{l}}\:\times\:100$$

### MTT cell viability studies

The MTT ((3-(4,5-Dimethylthiazol-2-yl)-2,5-Diphenyltetrazolium Bromide)) assay was employed to determine viability for the synthesized potent compounds **2b** and **2e**; the cell viability results portray the compounds’ cytotoxicity. The assay was conducted on RAW macrophage cells (RAW 264.7) with various dilutions of **2b** and **2e** (10, 20, 30, 40, and 50 µM). The results were confirmed with a control.

## Conclusion

In this work, the nicotinic acid scaffolds with the phenolic and amine derivatives were prepared in the presence of a base, 4-dimethylaminopyridine, and 1-(3-dimethylaminopropyl)-3-ethylcarbodiimide hydrochloride. We have developed and discovered potent amylase inhibitors **2b-k**. The compounds reported from this work were new pharmacophores. It has not been reported in the literature before. The synthesized inhibitors were screened and evaluated for their pharmacological activity. Developed inhibitors were investigated with in vitro biological studies such as antiradical (DPPH, ABTS cation radicals), anti-inflammation (induced human RBC hemolysis), and antidiabetic (α-amylase). Among all derivatives, the compounds **2b** and **2 h** (IC_50_ = 15.63 ± 0.13 µM and IC_50_ = 12.88 ± 0.19 µM (DPPH), IC_50_ = 19.89 ± 0.25 µM and 16.35 ± 0.25 µM (ABTS)) showed good radical scavenging efficiency compared with ascorbic acid (11.81 ± 0.04 µM (DPPH) and 11.90 ± 0.01 µM (ABTS)) as positive control and anti-inflammation activity was carried out using induced human RBC hemolysis in the presence of synthesized analogues **2b** (IC_50_ = 18.41 ± 0.13 µM) and **2e** (IC_50_ = 14.06 ± 0.15 µM) exhibited good inhibiting ability compared with the positive control ((ketorolac, (IC_50_ = 11.79 ± 0.17 µM)) and anti-hyperglycemic activity was evaluated using α-amylase enzyme with characterized inhibitors **2e** (IC_50_ = 1.324 ± 0.21 µg), and **2j** (IC_50_ = 1.326 ± 0.10 µg) exposed excellent inhibitory action while compared with acarbose drug (IC_50_ = 1.273 ± 0.12 µg). To explore the biological active molecule’s structure-activity relationship (SAR), this study proved that some of the designed and synthesized inhibitor molecules exhibited excellent inhibitory activities against in vitro targets. The in vitro and in silico approaches will be useful for future aspects such as lead optimization and developing novel building blocks of anti-hyperglycemic (α-amylase) inhibitors. This has provided us with a new avenue for future exploration.

## Supplementary Information

Below is the link to the electronic supplementary material.

Supplementary Material 1

## Data Availability

We declare that supporting data for this work is provided in the Supplementary Information. Raw and unprocessed nuclear magnetic resonance and other data are available from the corresponding author on reasonable request.
