# Supplementary material for: Discovery of new pyridine 3-carboxylic acid-based pharmacophores as dual anti-inflammatory and anti-hyperglycemic agents
Source: Sci Rep. 2025 Oct 3;15:34536. doi: 10.1038/s41598-025-17841-1 (PMC12494831; doi:10.1038/s41598-025-17841-1)
Supplement: Supplementary file 1 — Supplementary Material 1 [file 41598_2025_17841_MOESM1_ESM.docx]

**Discovery of new pyridine 3-carboxylic acid-based pharmacophores as dual anti-inflammatory and anti-hyperglycemic agents**

**Ramakrishnan K^a^, Lenin Nachimuthu^a^, Reshma Rajan^a^, Premkumar J^a^, Vallabh Mulay^a^, Meenakshi S^b^, Chandrakala A Narasimhulu^c^, Pragney Deme^d^, Sanjay Rajagopalan^e^, Ramanathan Lalgudi^f^, Akella Sivaramakrishna^a^, Karthikeyan S^a^, Rajagopal Desikan^a^***

*^a^ Department of Chemistry, School of Advanced Sciences, Vellore Institute of Technology, Vellore, Tamilnadu, India-632014.*

*^b^ Department of English, School of Social Sciences and Languages, Vellore Institute of Technology, Vellore, Tamilnadu, India-632014.*

*^c^ Division of Metabolic and Cardiovascular Sciences, Burnett School Of Biomedical Sciences, University Of Central Florida, Orlando, USA.*

*^d^ Department of Neurology, The Johns Hopkins University School of Medicine, Baltimore, MD, USA.*

*^e^ Department of Medicine, School of Medicine, Case Western Reserve University, Cleveland, Ohio, USA.*

*^f^ Aries Science & Technology, Columbus, Ohio, USA.*

**Corresponding author email ID:* [*rajagopal.desikan@vit.ac.in*](mailto:rajagopal.desikan@vit.ac.in) *(Rajagopal D)*

**Chemical characterization details:**


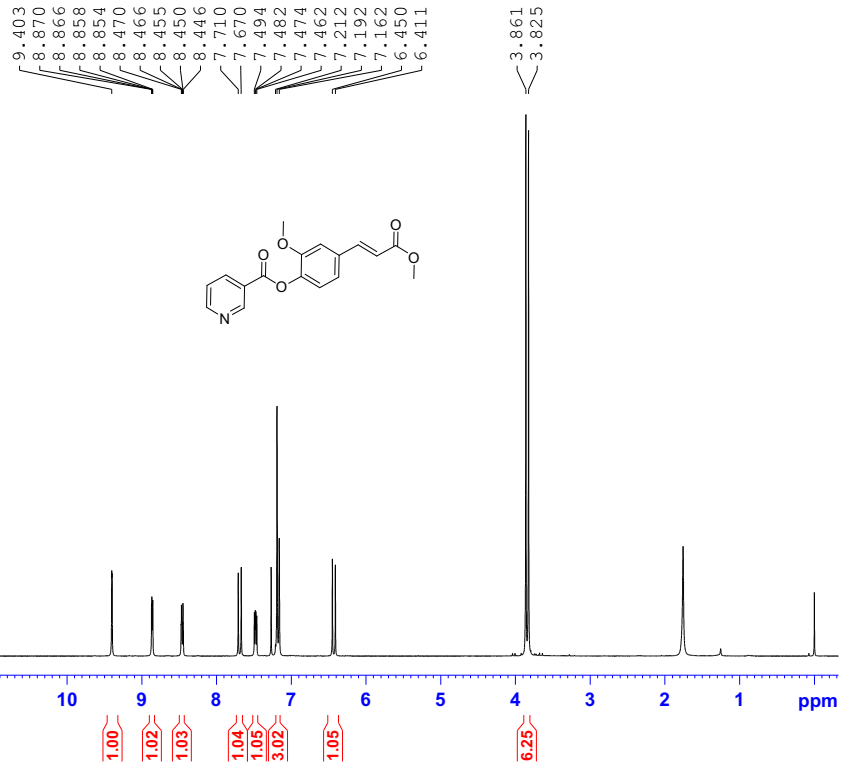


Figure S1. ^1^H NMR of 2-methoxy-4-(3-methoxy-3-oxoprop-1-en-1-yl)phenyl nicotinate (2b)


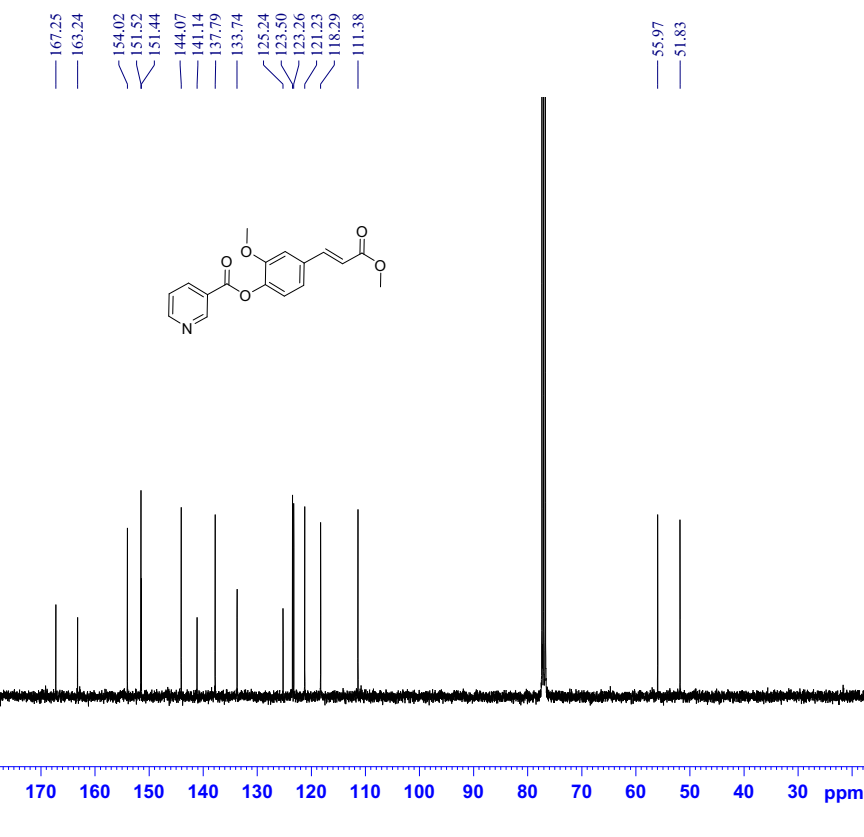


Figure S2. ^13^C NMR of 2-methoxy-4-(3-methoxy-3-oxoprop-1-en-1-yl)phenyl nicotinate (2b)


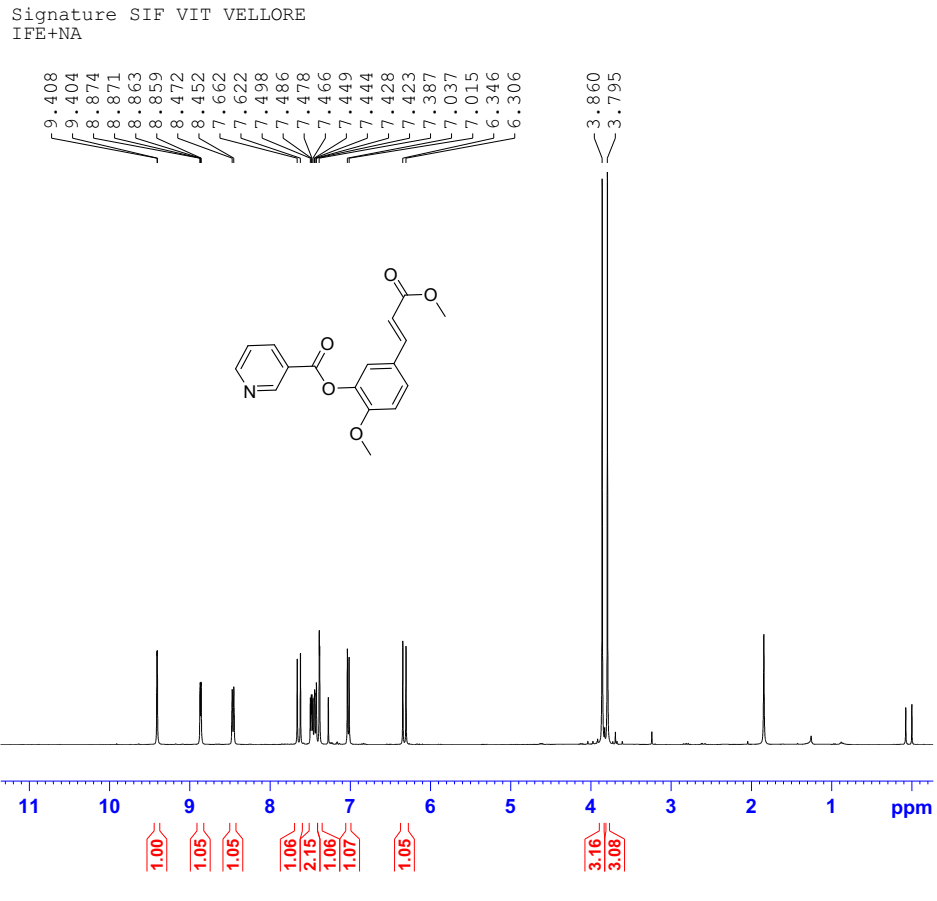


Figure S3. ^1^H NMR of 2-methoxy-5-(3-methoxy-3-oxoprop-1-en-1-yl)phenyl nicotinate (2c)


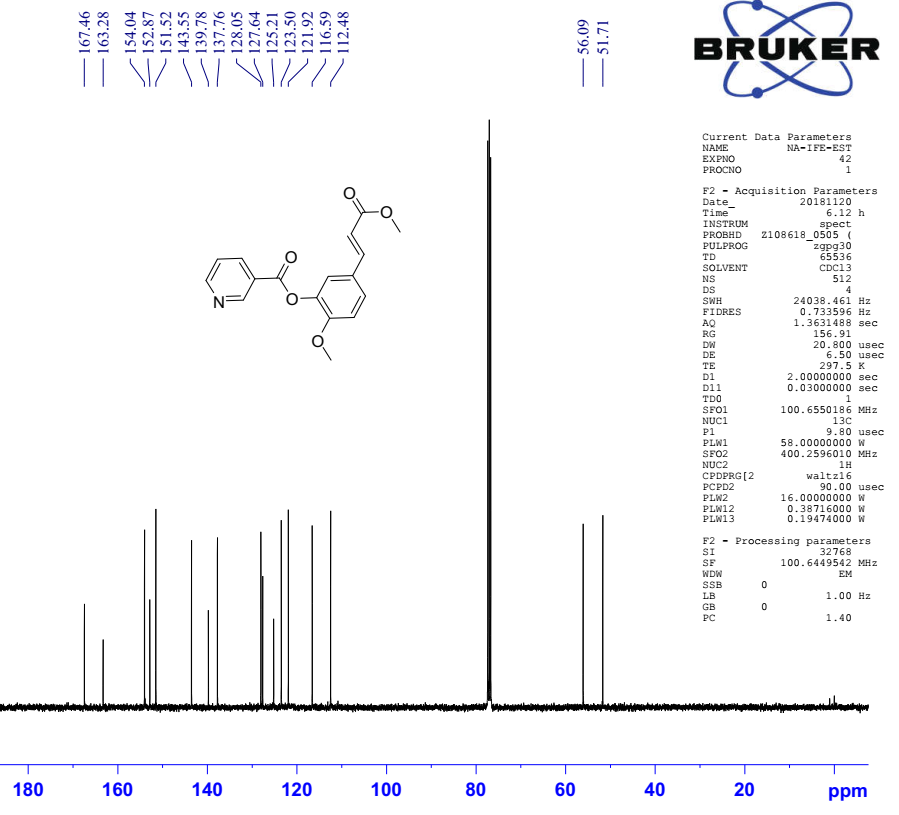


Figure S4. ^13^C of 2-methoxy-5-(3-methoxy-3-oxoprop-1-en-1-yl)phenyl nicotinate (2c)


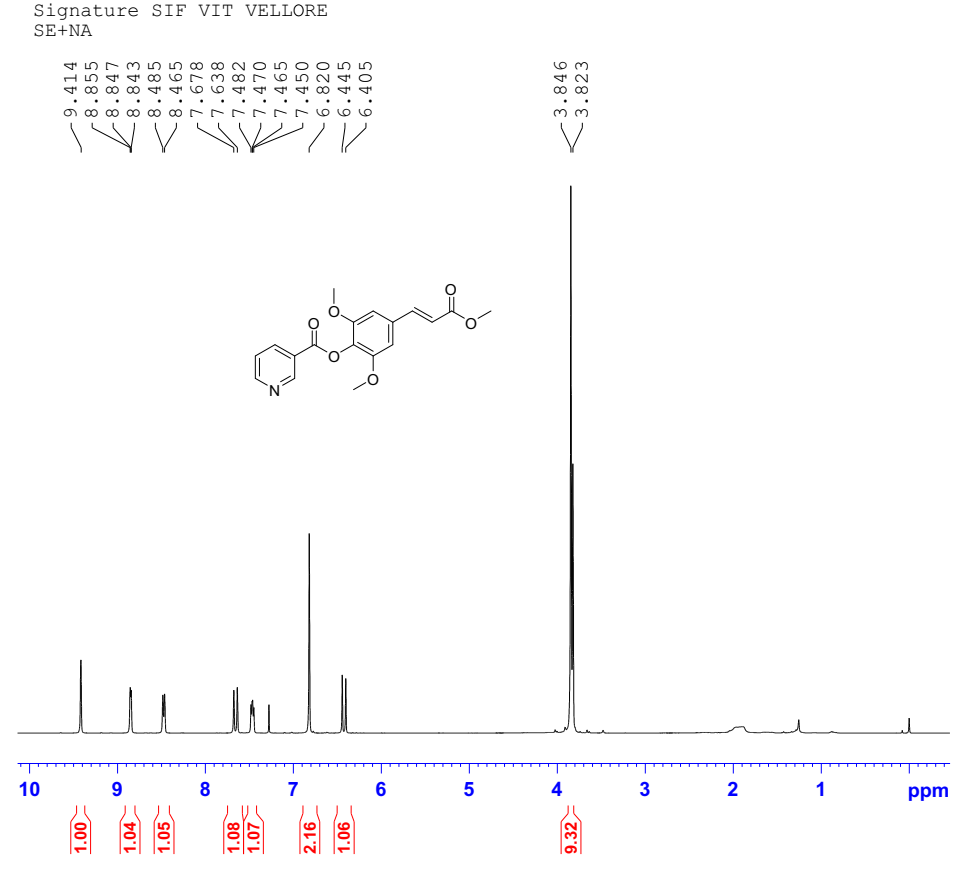


Figure S5. ^1^H NMR of 2,6-dimethoxy-4-(3-methoxy-3-oxoprop-1-en-1-yl)phenyl nicotinate (2d)


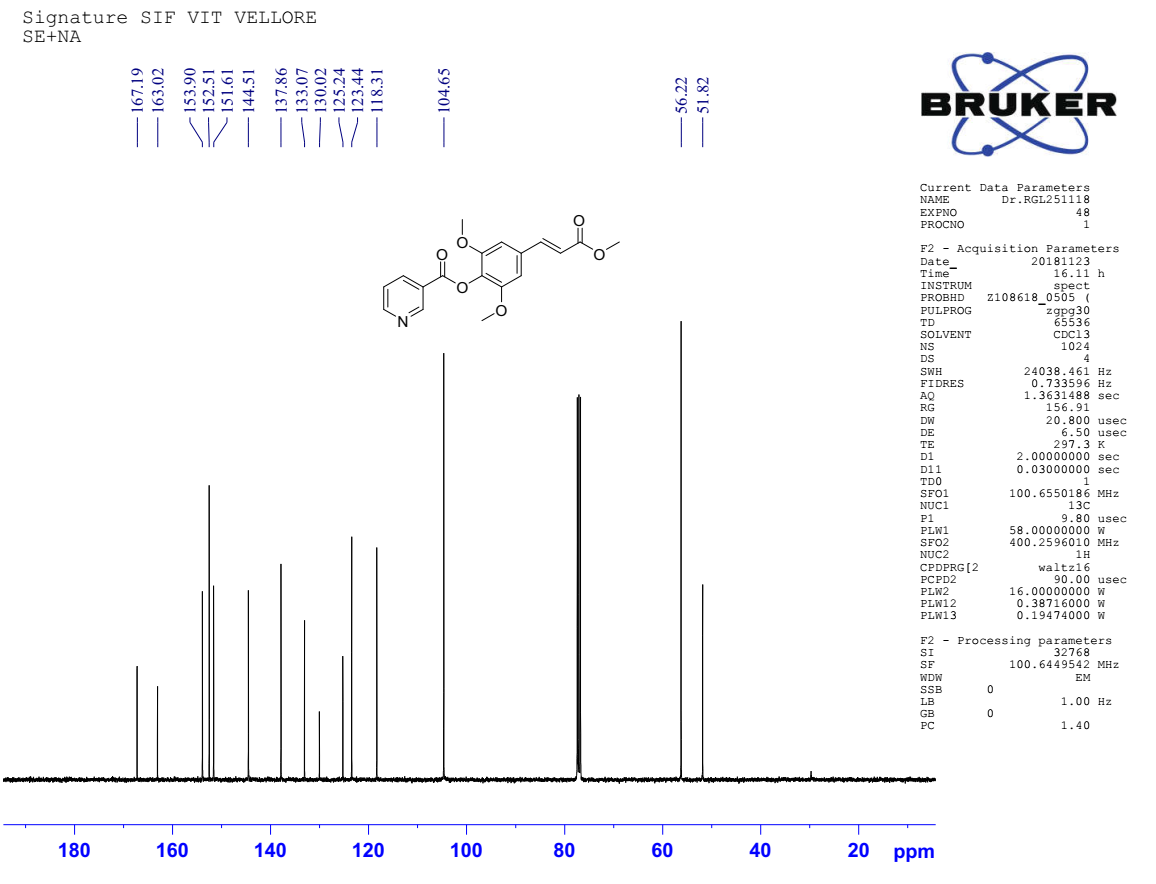


Figure S6. ^13^C NMR of 2,6-dimethoxy-4-(3-methoxy-3-oxoprop-1-en-1-yl)phenyl nicotinate (2d)


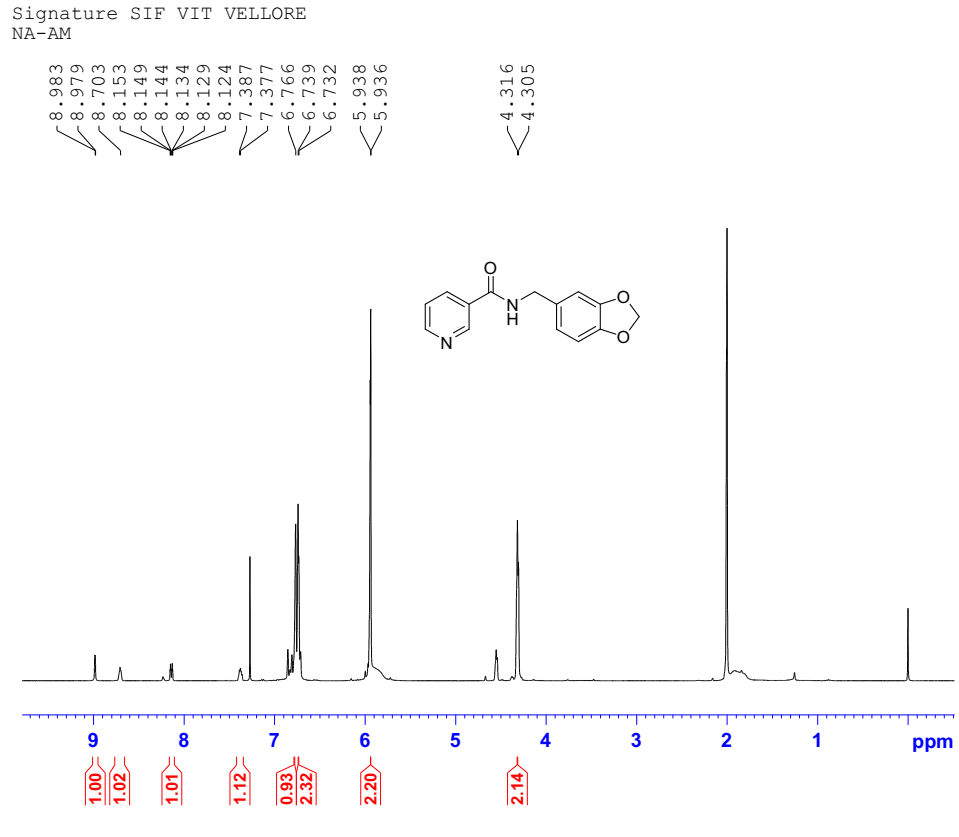


Figure S7. ^1^H NMR of N-(benzo[1,3]dioxol-5-ylmethyl)nicotinamide (2e)


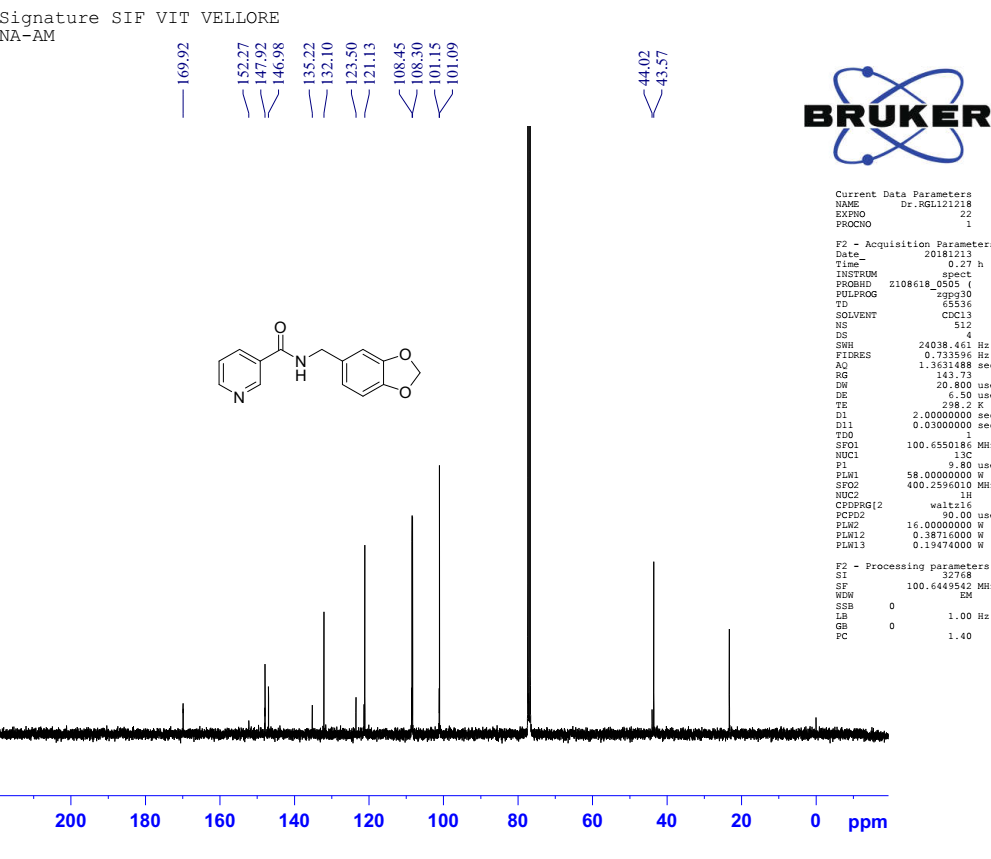


Figure S8. ^13^C NMR of N-(benzo[1,3]dioxol-5-ylmethyl)nicotinamide (2e)


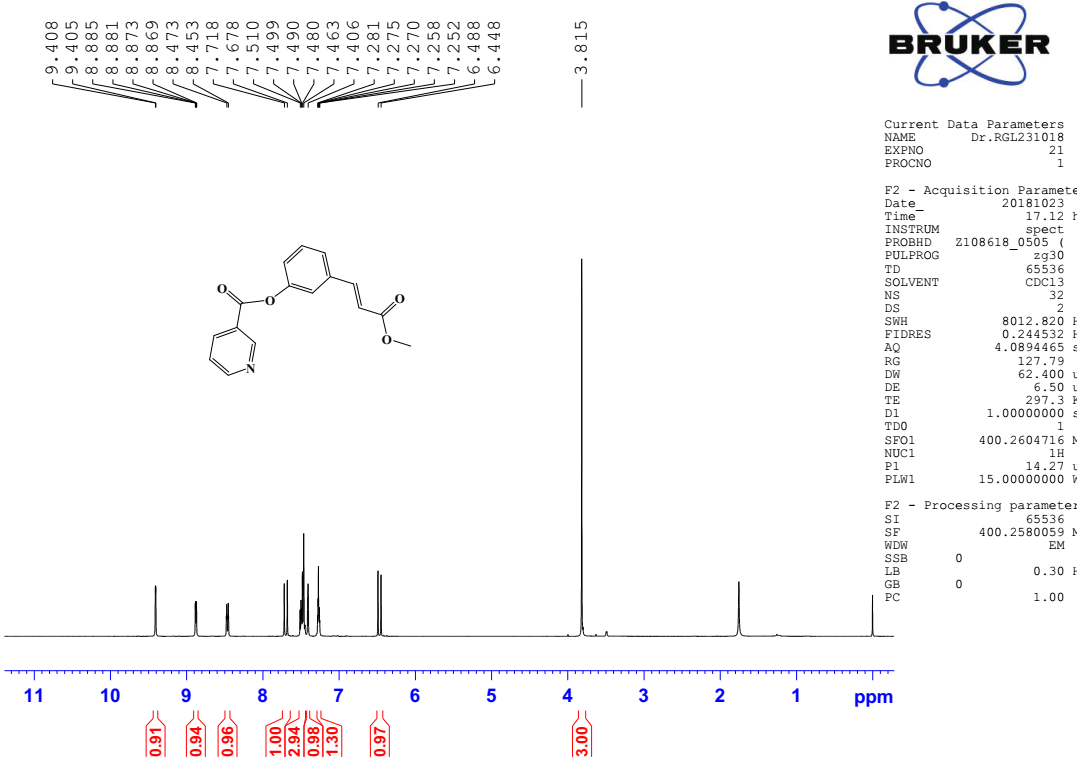


Figure S9. ^1^H NMR of 3-(3-methoxy-3-oxoprop-1-en-1-yl)phenyl nicotinate (2f)


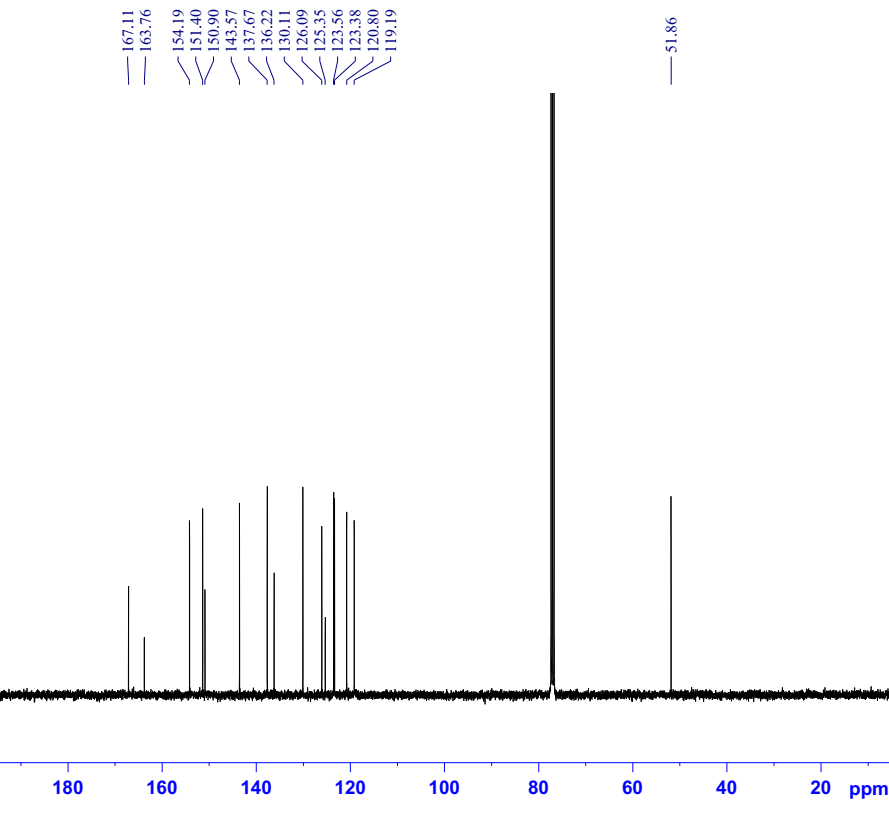


Figure S10. ^13^C NMR of 3-(3-methoxy-3-oxoprop-1-en-1-yl)phenyl nicotinate (2f)


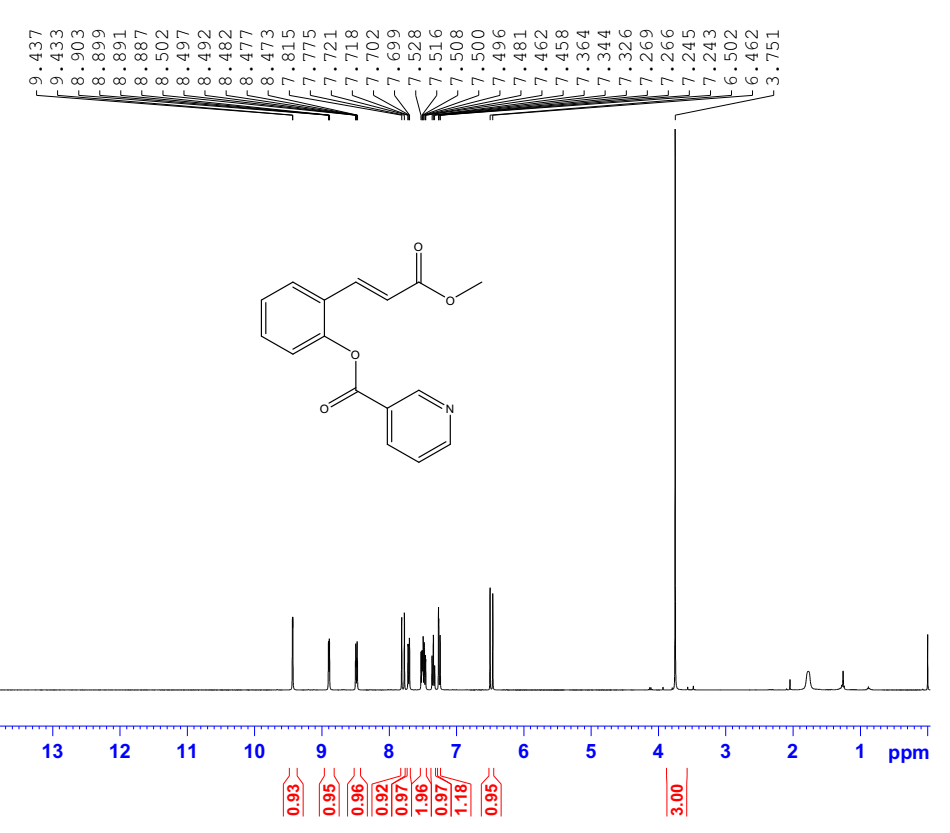


Figure S11. ^1^H NMR of 2-(3-methoxy-3-oxoprop-1-en-1-yl)phenyl nicotinate (2g)


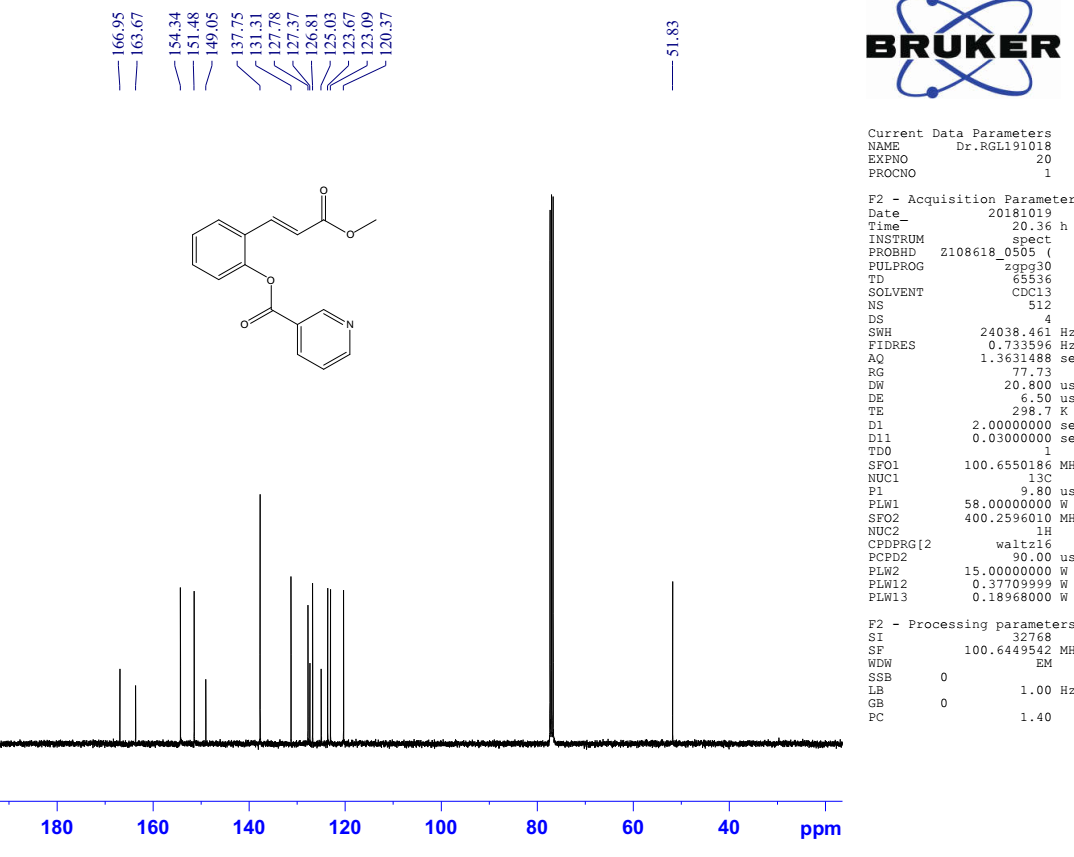


Figure S12. ^13^C NMR of 2-(3-methoxy-3-oxoprop-1-en-1-yl)phenyl nicotinate (2g)


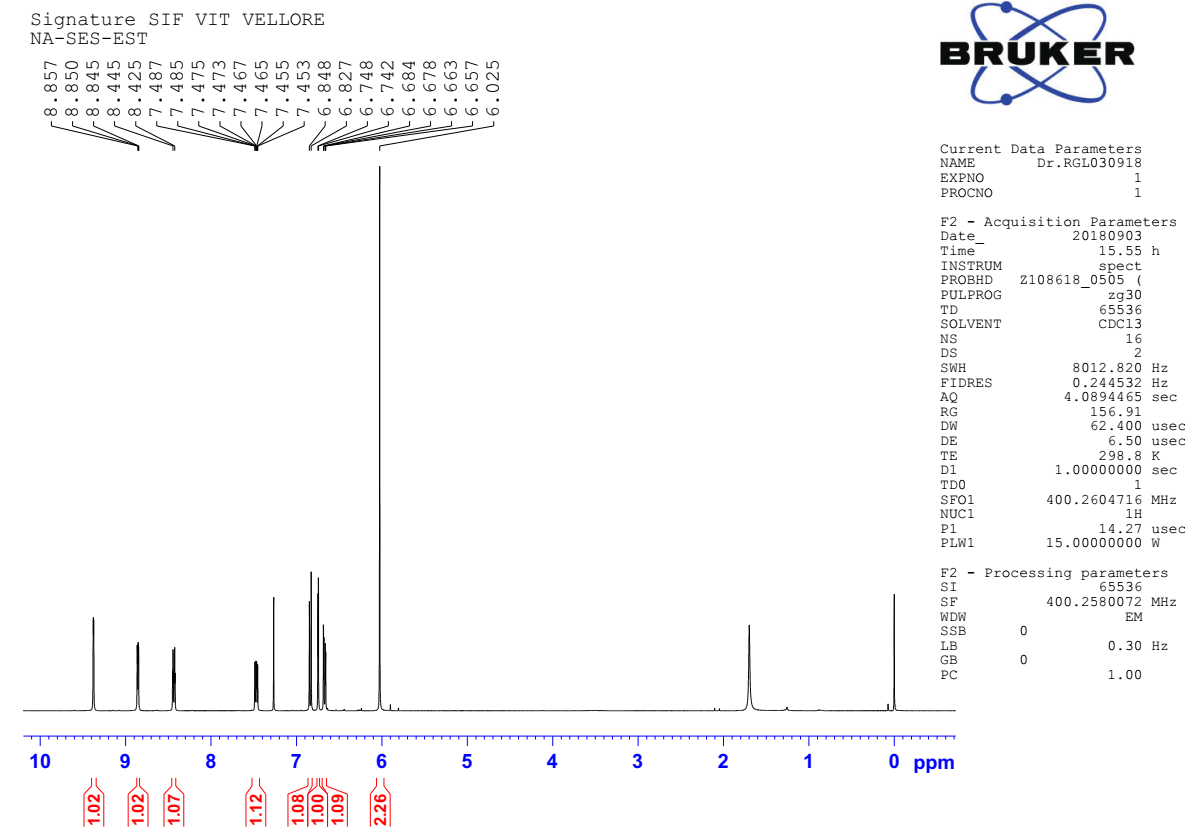


Figure S13. 1H NMR of benzo[1,3]dioxol-5-yl nicotinate (2h)


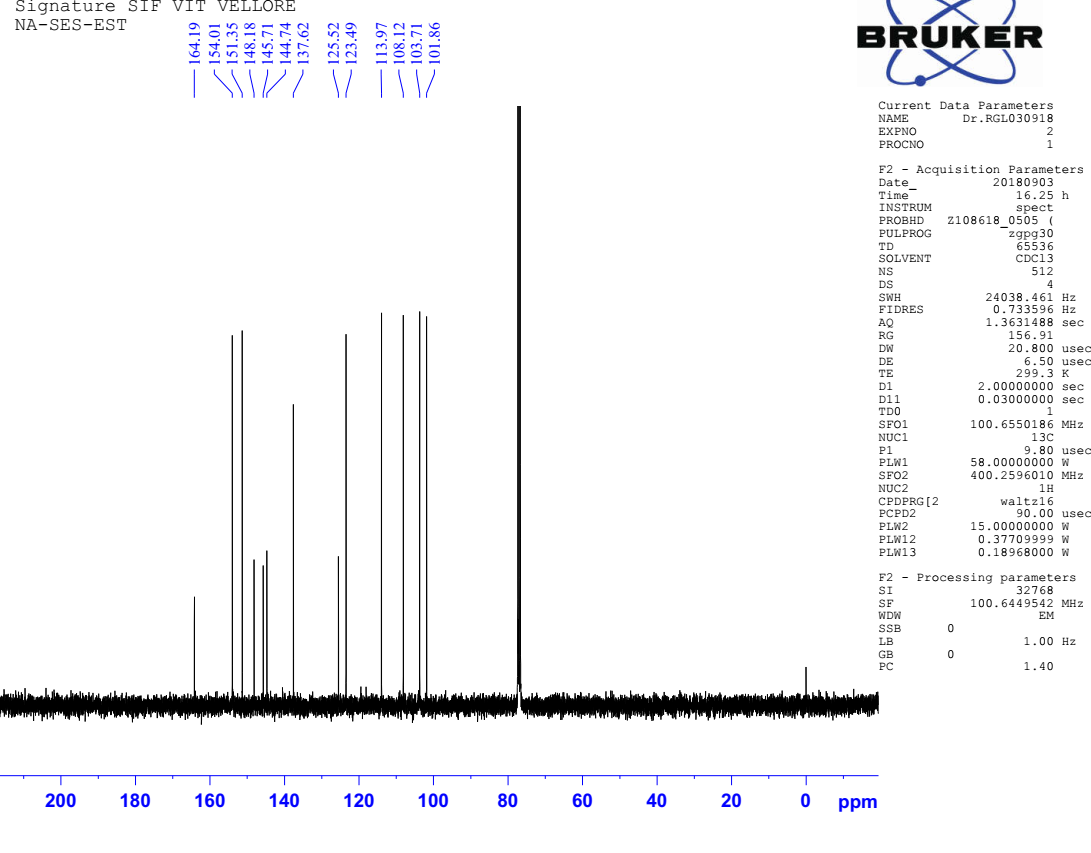


Figure S14. ^13^C NMR of benzo[1,3]dioxol-5-yl nicotinate (2h)


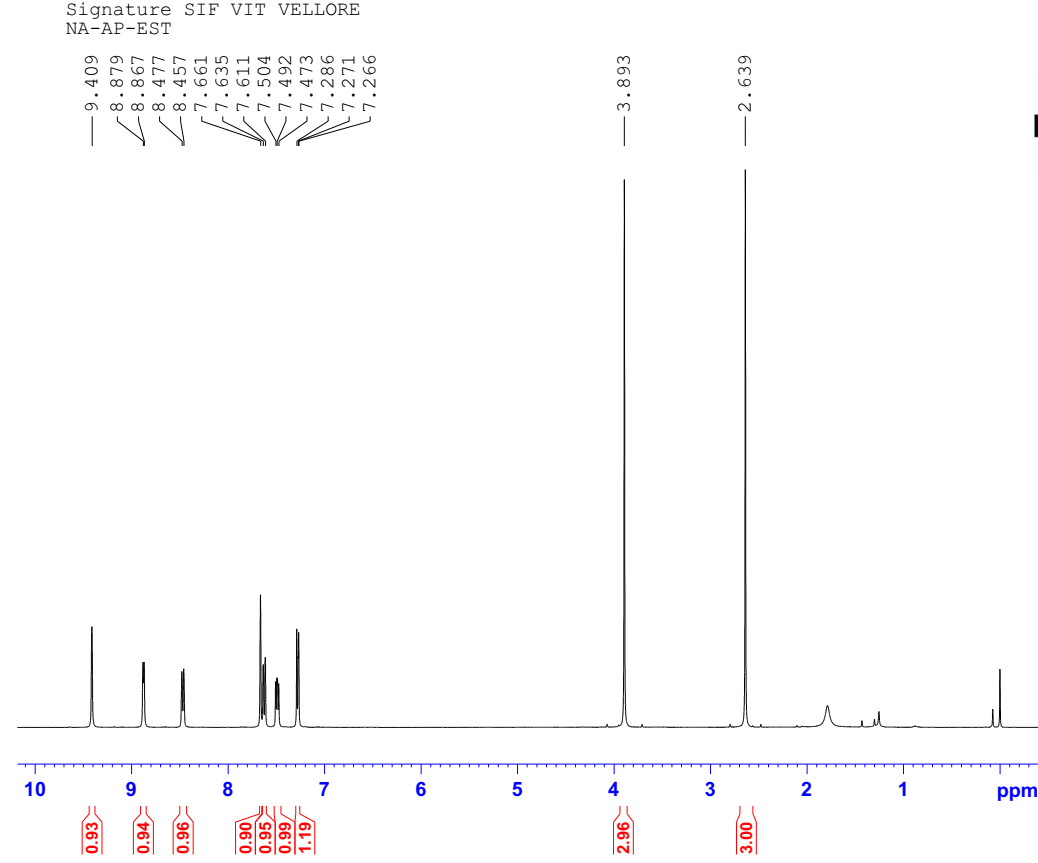


Figure S15. ^1^H NMR of 4-acetyl-2-methoxyphenyl nicotinate (2i)


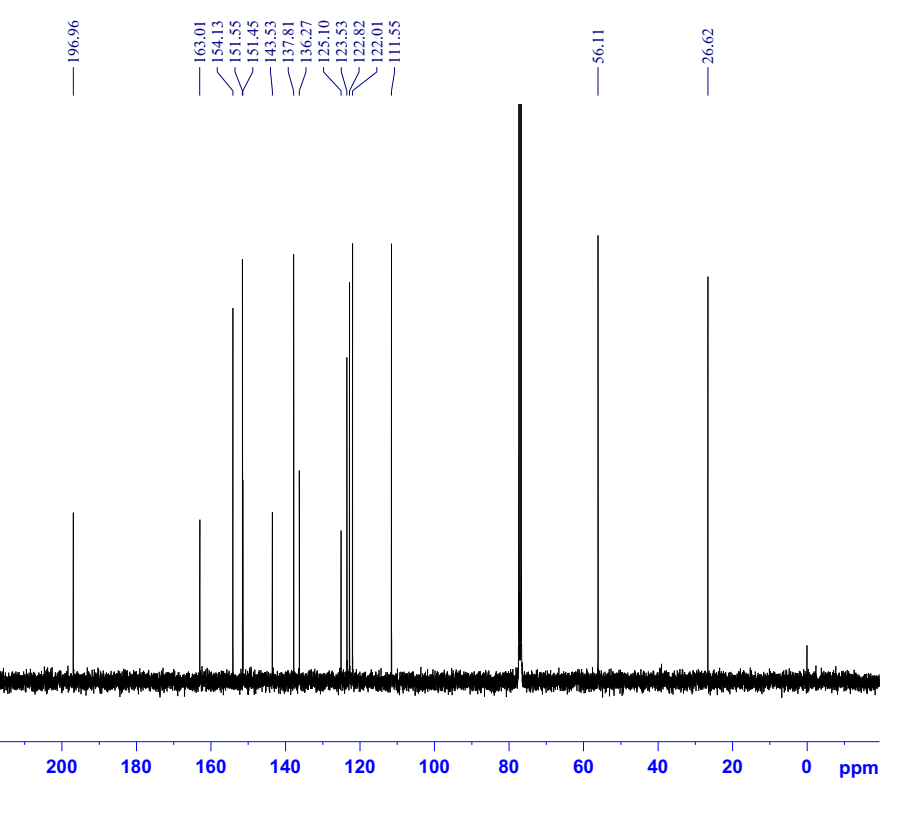


Figure S16. ^13^C NMR of 4-acetyl-2-methoxyphenyl nicotinate (2i)


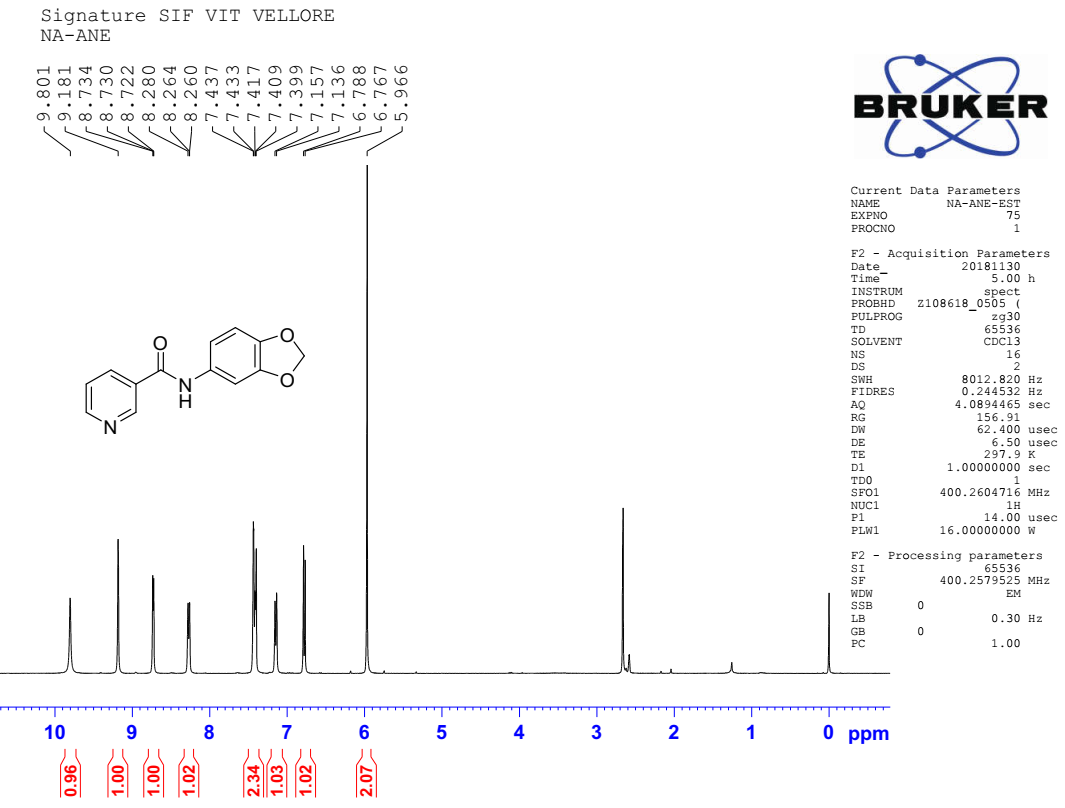


Figure S17. ^1^H NMR of N-(benzo[1,3]dioxol-5-yl)nicotinamide (2j)


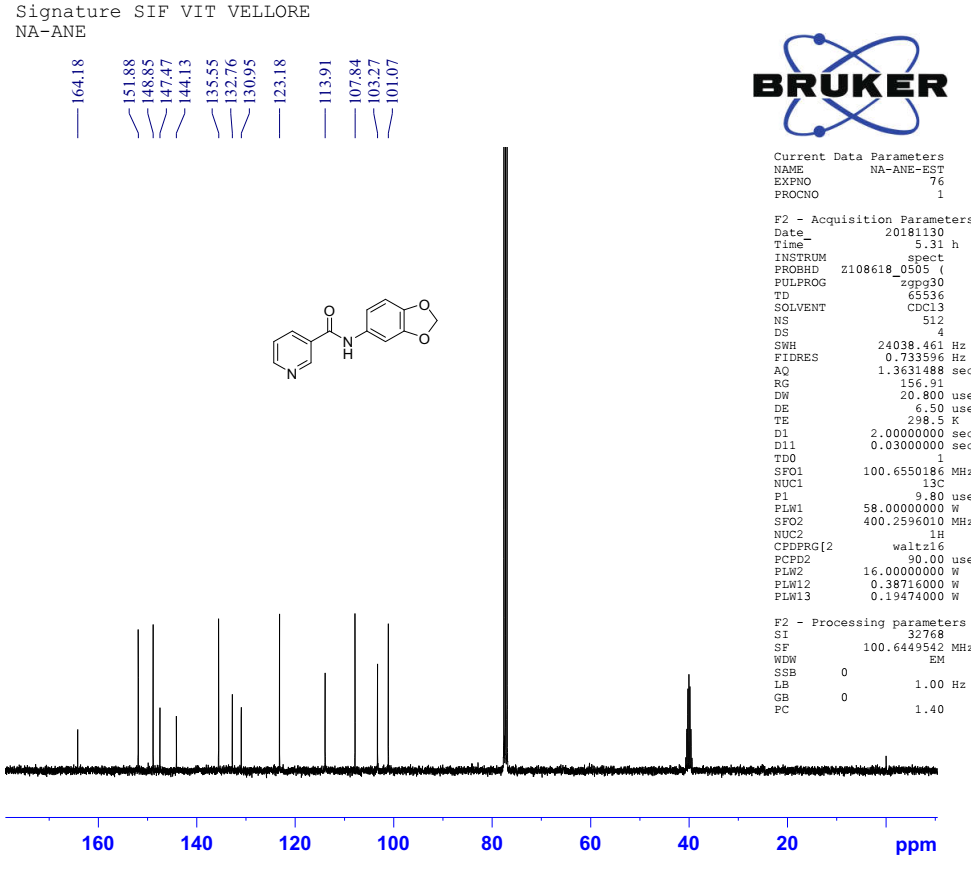


Figure S18. ^13^C NMR of N-(benzo[1,3]dioxol-5-yl)nicotinamide (2j)


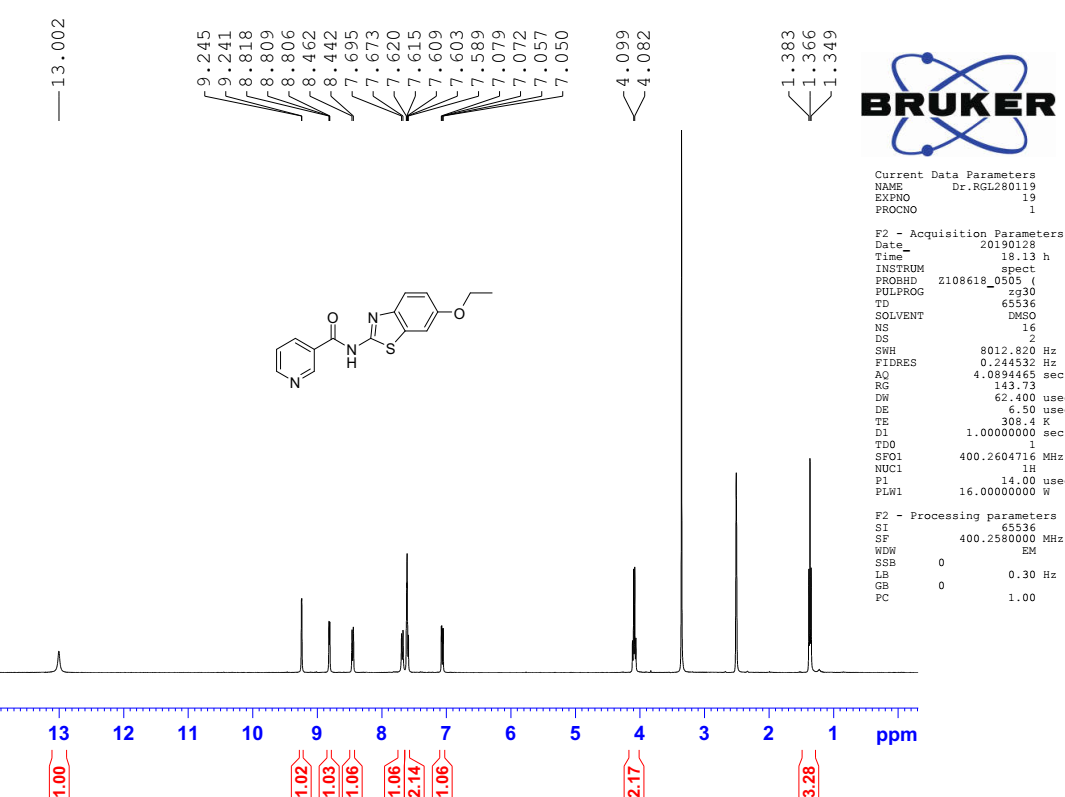


Figure S19. ^1^H NMR of N-(6-ethoxybenzothiazol-2-yl)nicotinamide (2k)


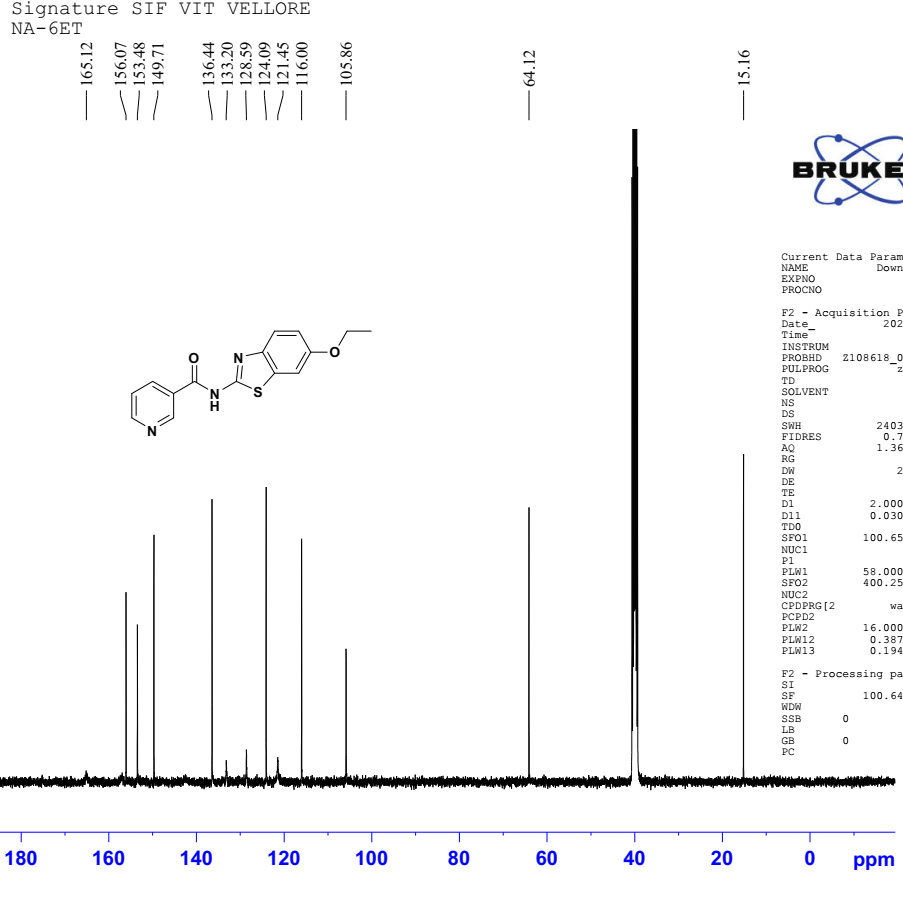


Figure S20. ^13^C NMR of N-(6-ethoxybenzothiazol-2-yl)nicotinamide (2k)

Figure S21. FT-IR spectrum of 2-methoxy-4-(3-methoxy-3-oxoprop-1-en-1-yl)phenyl nicotinate (2b)

Figure S22. FT-IR spectrum of 2-methoxy-5-(3-methoxy-3-oxoprop-1-en-1-yl)phenyl nicotinate (2c)

Figure S23. FT-IR spectrum of 2,6-dimethoxy-4-(3-methoxy-3-oxoprop-1-en-1-yl)phenyl nicotinate (2d)

Figure S24. FT-IR spectrum of N-(benzo[1,3]dioxol-5-ylmethyl)nicotinamide (2e)

Figure S25. FT-IR spectrum of 3-(3-methoxy-3-oxoprop-1-en-1-yl)phenyl nicotinate (2f)

Figure S26. FT-IR spectrum of 2-(3-methoxy-3-oxoprop-1-en-1-yl)phenyl nicotinate (2g)

Figure S27. FT-IR spectrum of benzo[1,3]dioxol-5-yl nicotinate (2h)

FigureS28. FT-IR spectrum of 4-acetyl-2-methoxyphenyl nicotinate (2i)

Figure S29. FT-IR spectrum of N-(benzo[1,3]dioxol-5-yl)nicotinamide (2j)

Figure S30. FT-IR spectrum of N-(6-ethoxybenzothiazol-2-yl)nicotinamide (2k)


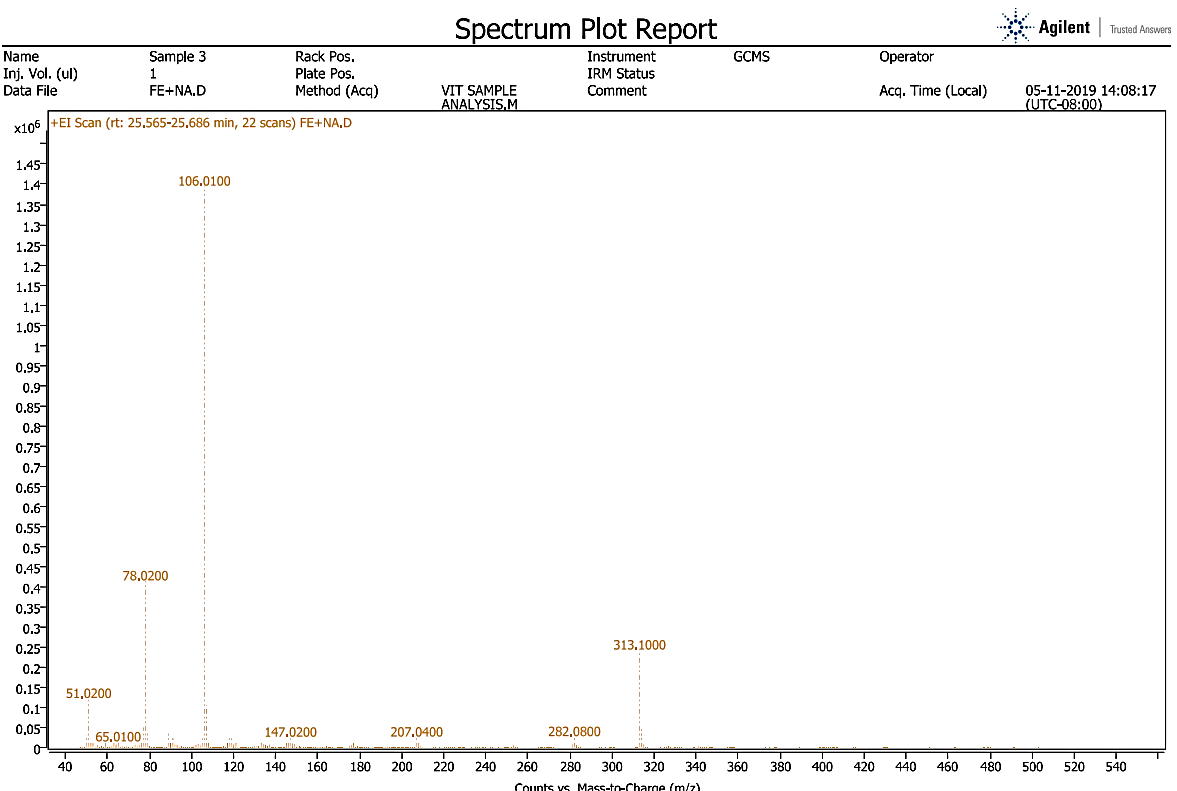


Figure S31. GC-MS spectrum of 2-methoxy-4-(3-methoxy-3-oxoprop-1-en-1-yl)phenyl nicotinate (2b)


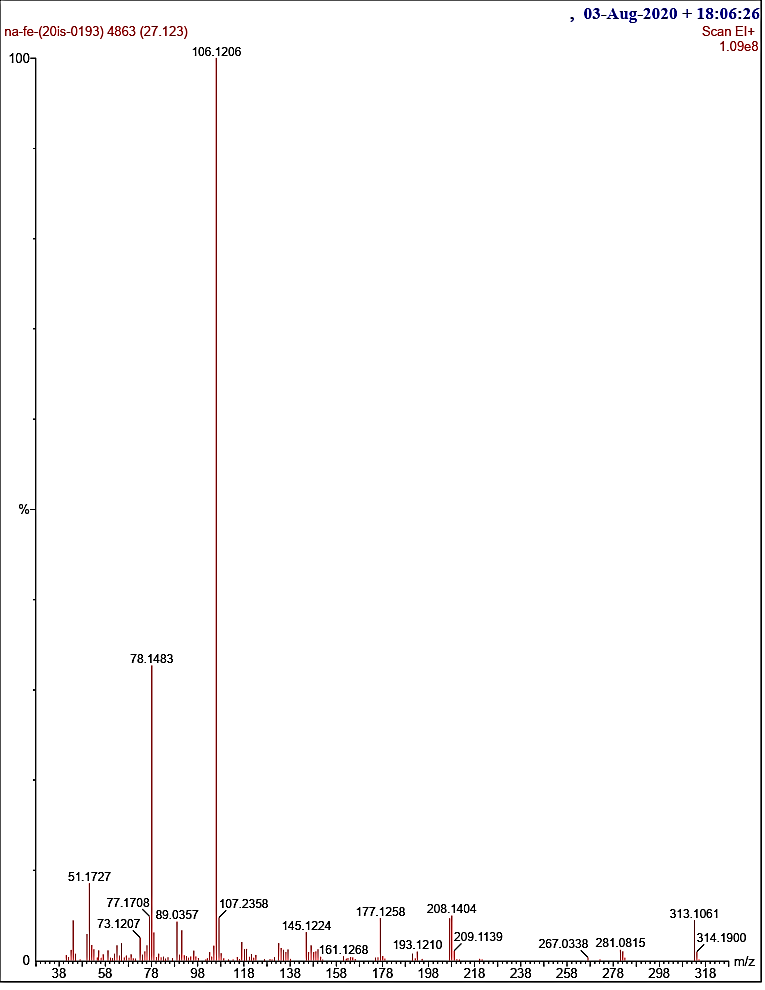


Figure S32. GC-MS spectrum of 2-methoxy-5-(3-methoxy-3-oxoprop-1-en-1-yl)phenyl nicotinate (2c)


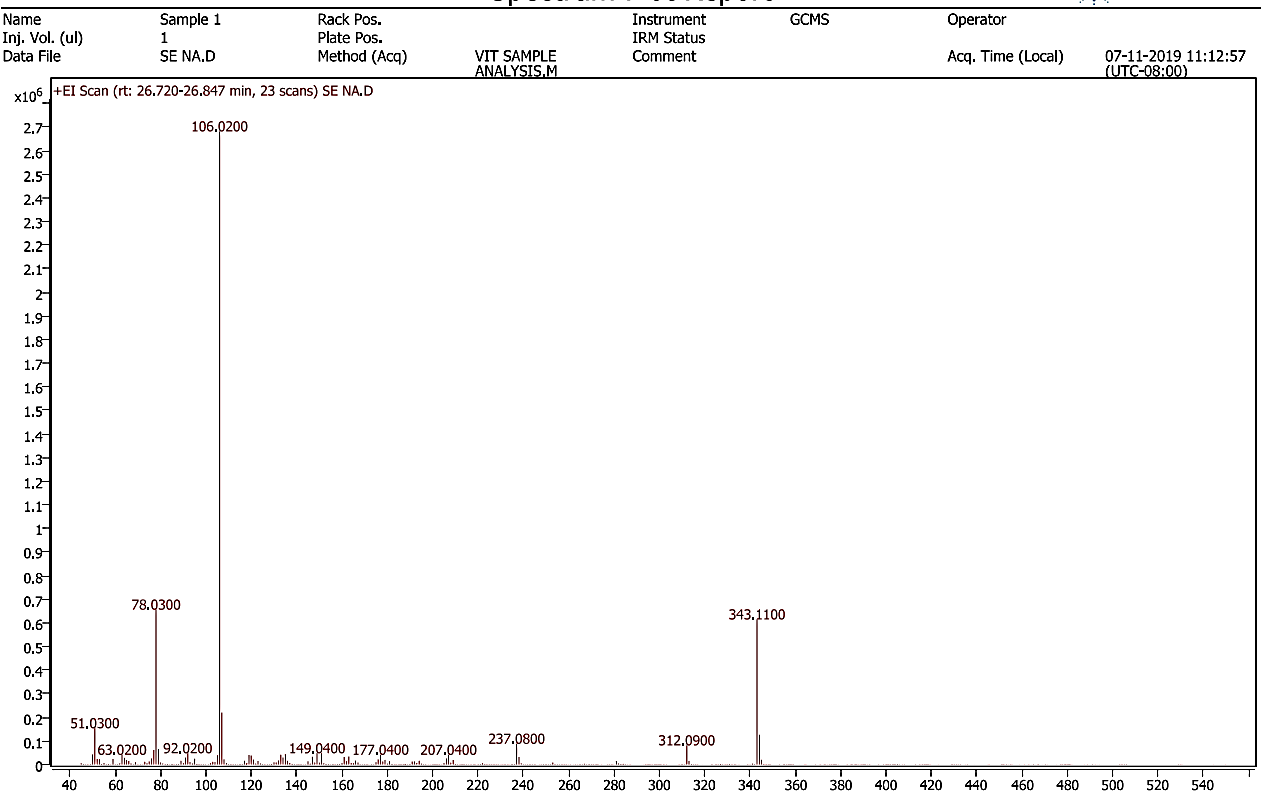


Figurer S33. GC-MS spectrum of 2,6-dimethoxy-4-(3-methoxy-3-oxoprop-1-en-1-yl)phenyl nicotinate (2d)


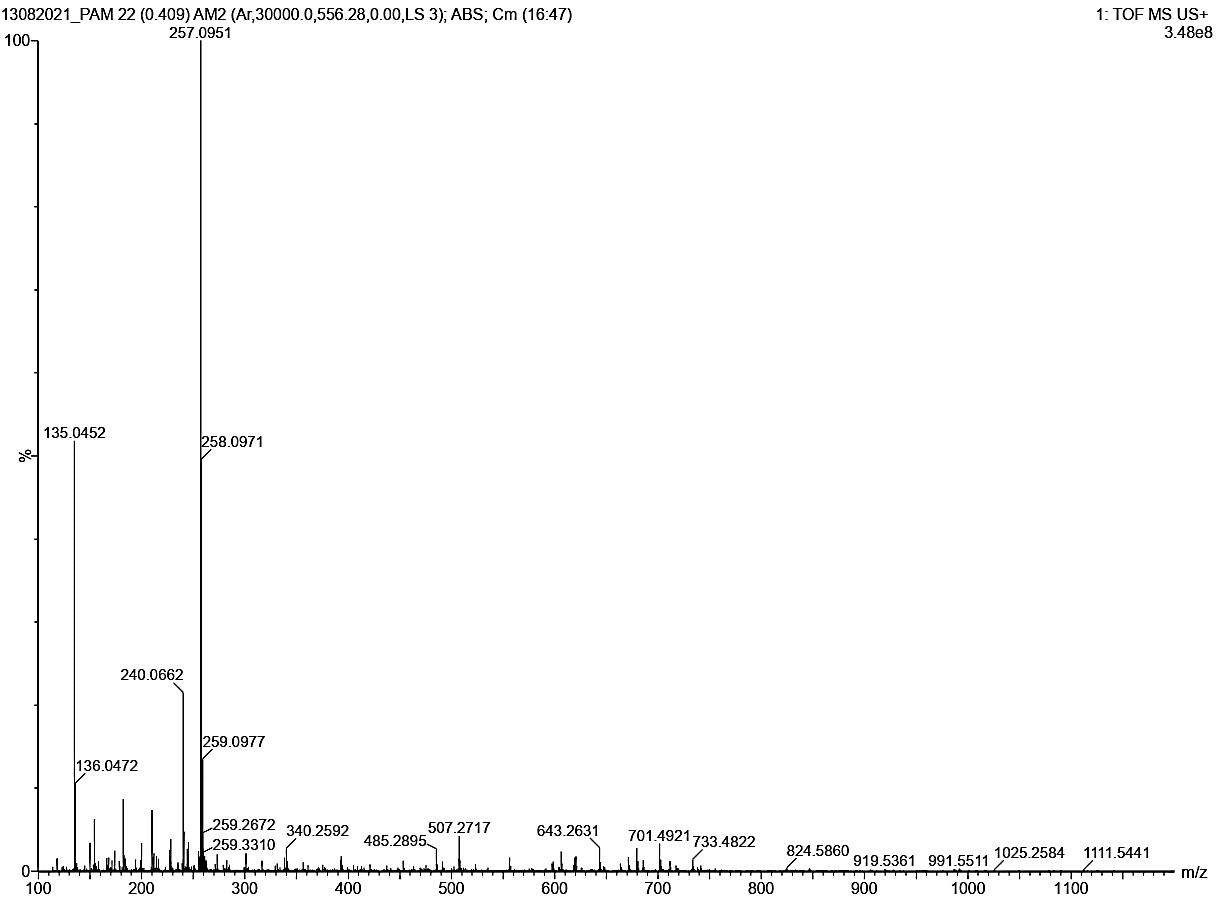


Figure S34. HRMS spectrum of N-(benzo[1,3]dioxol-5-ylmethyl)nicotinamide (2e)


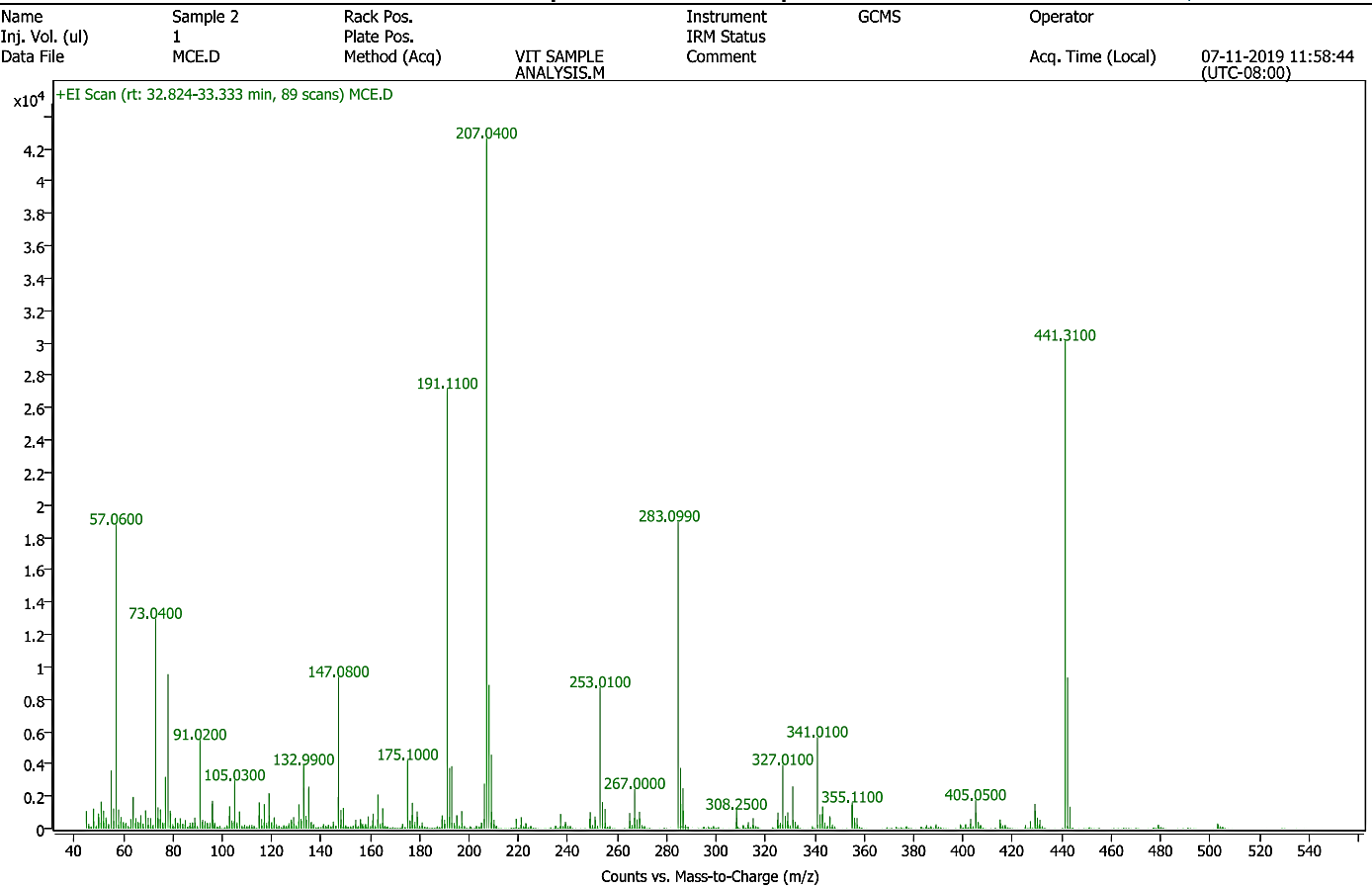


Figure S35. GC-MS spectrum of 3-(3-methoxy-3-oxoprop-1-en-1-yl)phenyl nicotinate (2f)


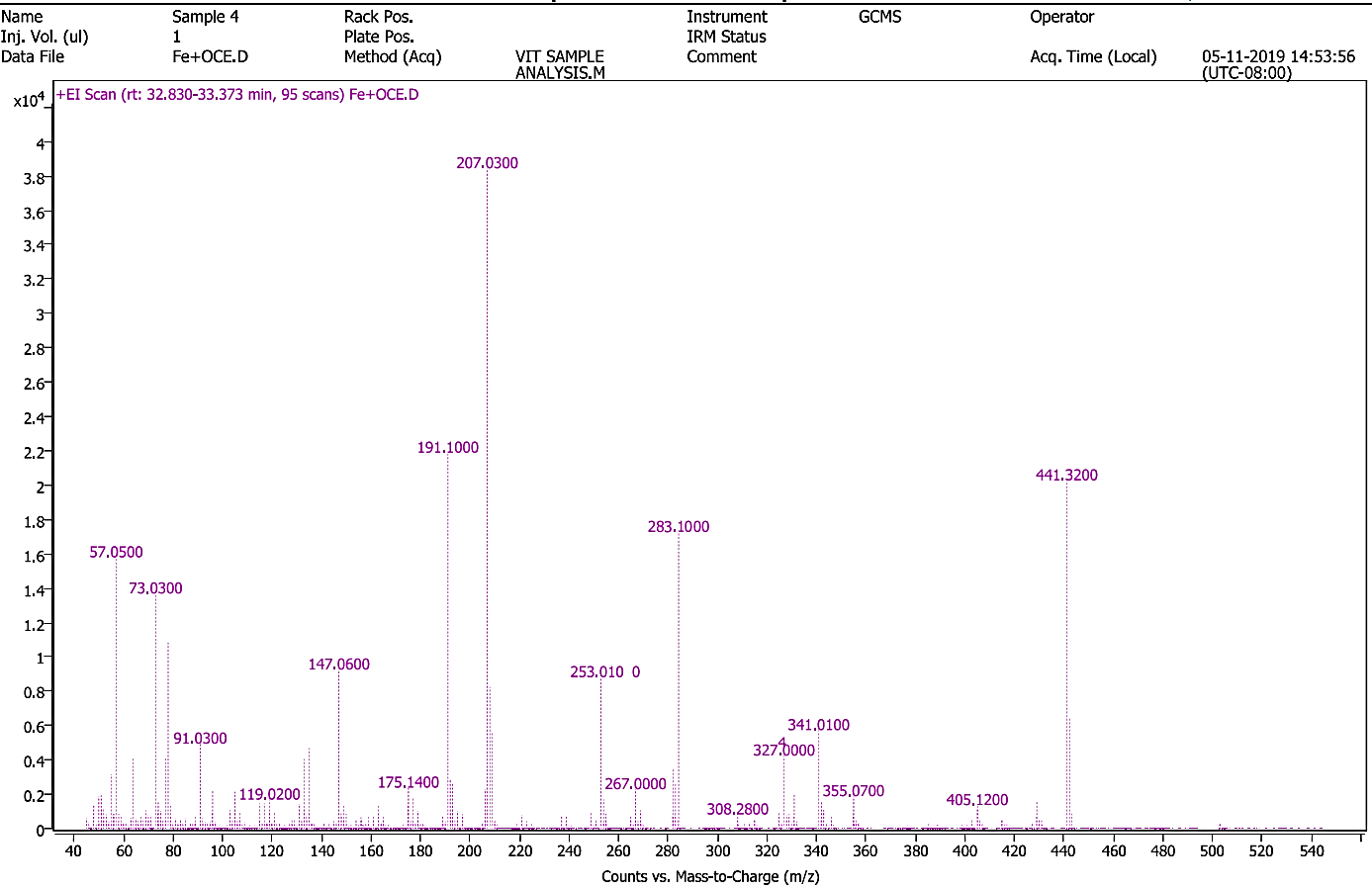


Figure S36. GC-MS spectrum of 2-(3-methoxy-3-oxoprop-1-en-1-yl)phenyl nicotinate (2g)


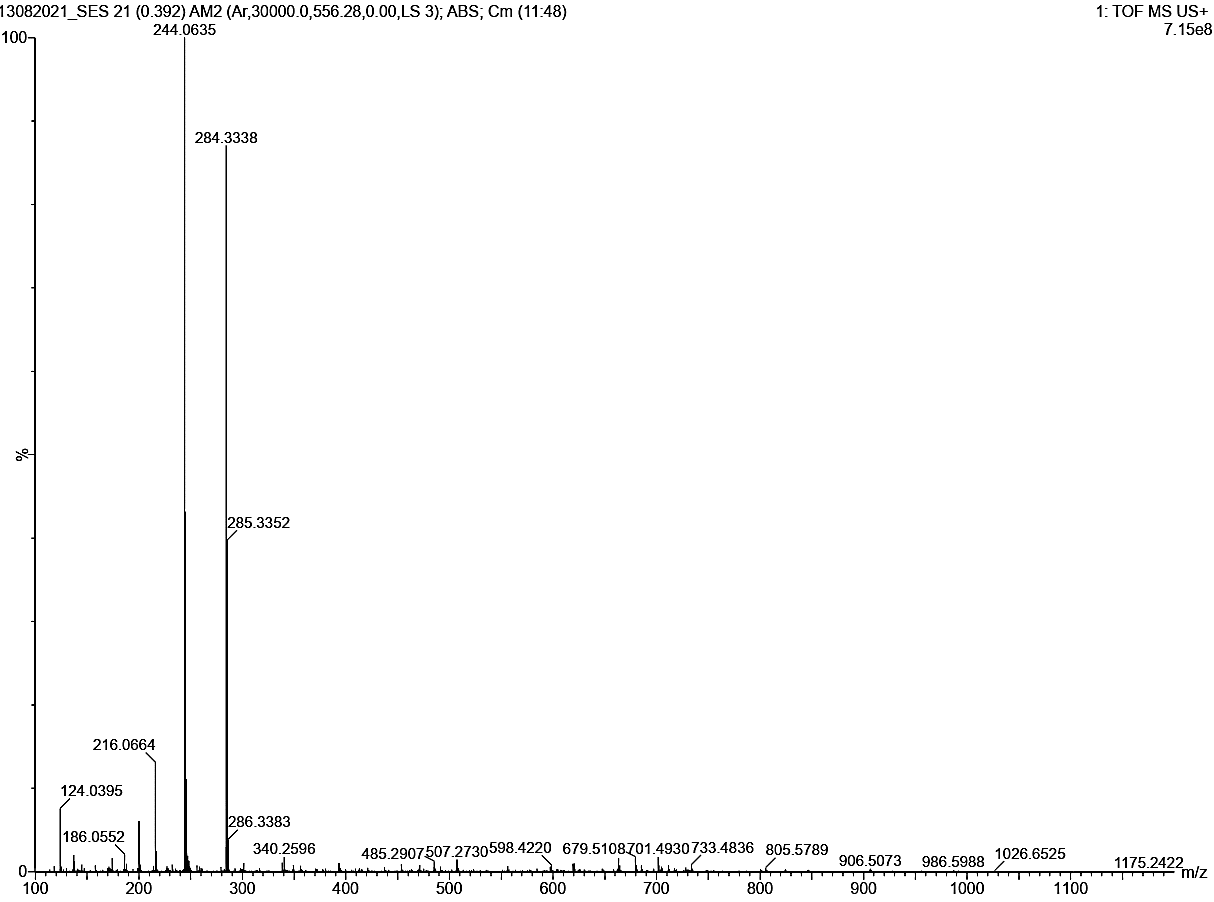


Figure S37. HRMS spectrum of benzo[1,3]dioxol-5-yl nicotinate (2h)


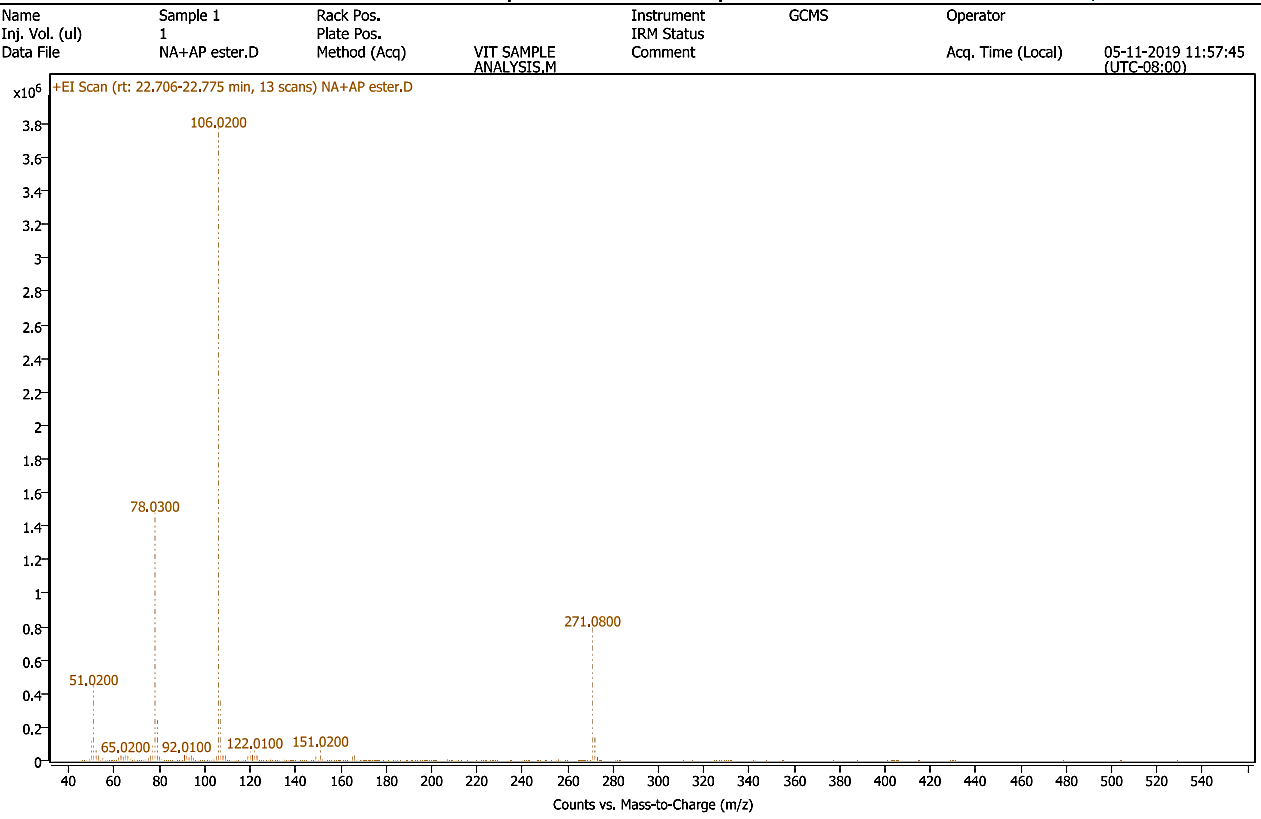


Figure S38. GC-MS spectrum of 4-acetyl-2-methoxyphenyl nicotinate (2i)


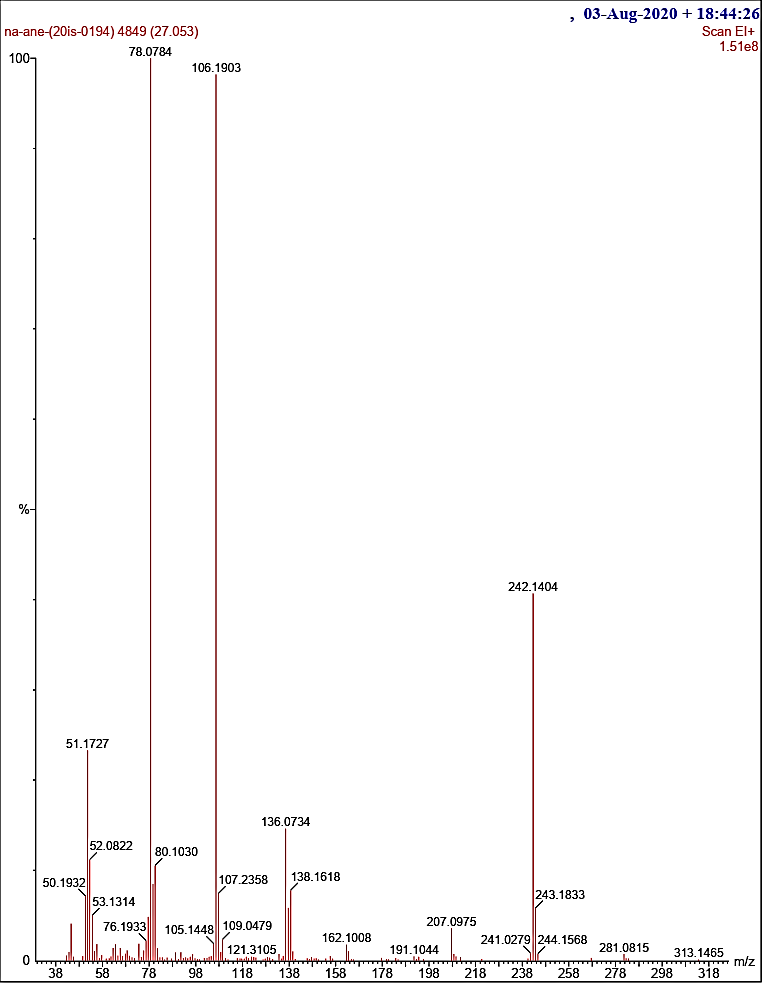


Figure S39. GC-MS spectrum of N-(benzo[1,3]dioxol-5-yl)nicotinamide (2j)


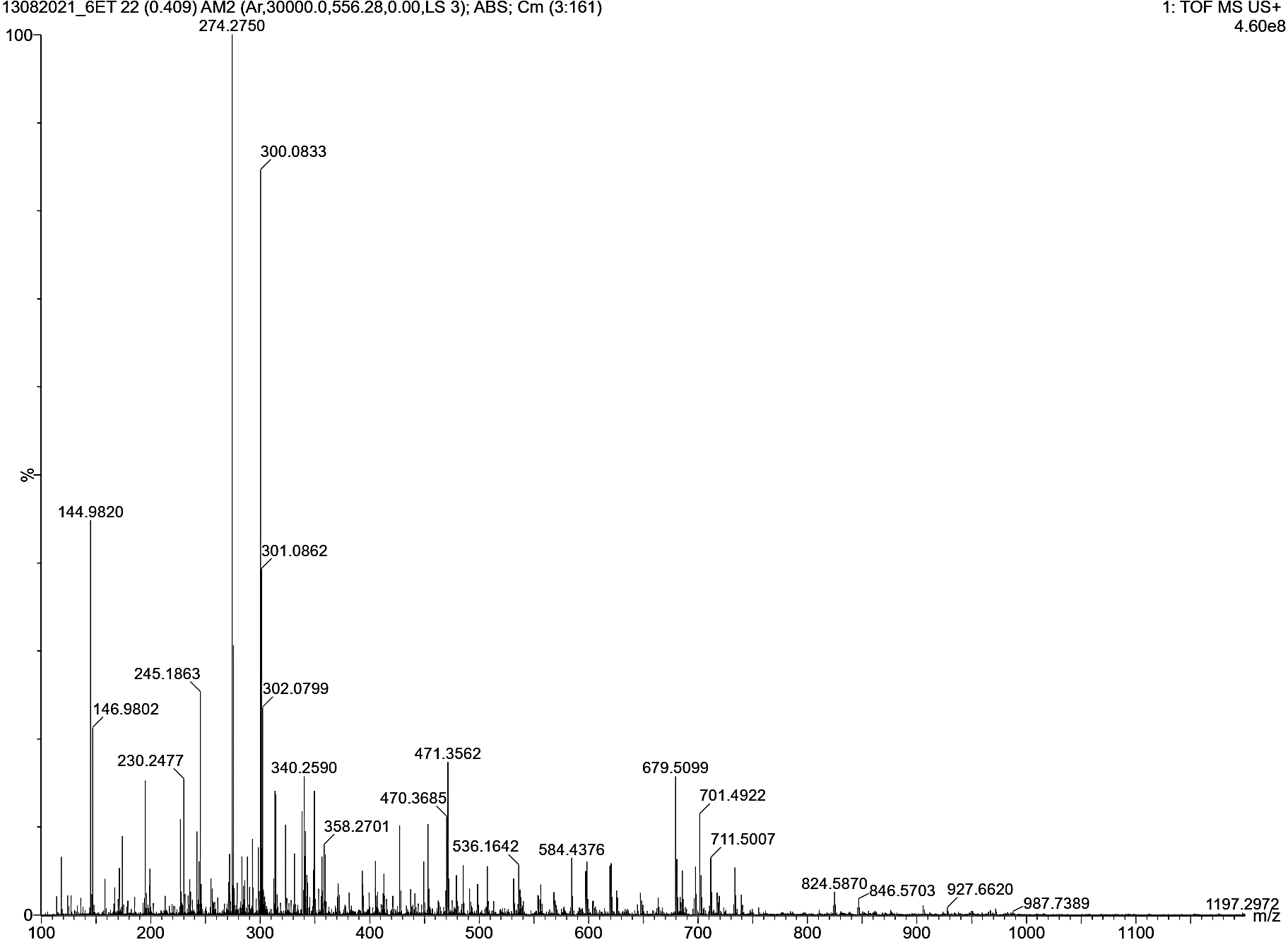


Figure S40. HRMS spectrum of N-(6-ethoxybenzothiazol-2-yl)nicotinamide (2k)

Table S1 Experimental bond length, bond angle, and torsion angle

| **Parameter** | **Compound 2d** | **Compound 2k** | | **Compound 2d** |  |
| --- | --- | --- | --- | --- | --- |
| **Bond length** |  |  |  | **Torsion angle** |  |
| C3-N1 | 1.360 | S1-C8 | 1.740 | C4-C5-C6-O2 | 179.38 |
| C4-N1 | 1.374 | O1-C6 | 1.217 | C6-C5-C7-O1 | 177.07 |
| C6-O1 | 1.203 | O2-C14 | 1.416 | C5-C6-O2-C7 | 169.73 |
| C6-O2 | 1.360 | N1-C5 | 1.337 | C15-C16-C17-O6 | 174.99 |
| O2-C5 | 1.371 | N2-C7 | 1.381 | C16-C17-O6-C18 | 178.32 |
| O3-C7 | 1.229 | N3-C9 | 1.391 |  |  |
| O4-C17 | 1.425 | S1-C8 | 1.740 |  |  |
| O5-C18 | 1.421 | O1-C6 | 1.217 |  |  |
|  |  | S1-C7 | 1.746 |  |  |
|  |  | O2-C11 | 1.368 |  |  |
|  |  | N1-C4 | 1.335 |  |  |
|  |  | N2-C6 | 1.367 |  |  |
|  |  | N3-C7 | 1.293 |  |  |
| **Bond angle** |  |  |  |  |  |
| C3-N1-C4 | 116.7 | C8-S1-C7 | 87.94 |  |  |
| C6-O2-C7 | 117.1 | C4-N1-C5 | 116.65 |  |  |
| O1-C6-O2 | 122.3 | C7-N3-C9 | 109.67 |  |  |
| C12-O3-C13 | 117.5 | N1-C4-C3 | 123.92 |  |  |
| C8-O4-C14 | 117.6 | O1-C6-N2 | 121.75 |  |  |
| C17-O6-C18 | 117.4 | N2-C6-C1 | 115.64 |  |  |
|  |  | N3-C7-S1 | 117.18 |  |  |
|  |  | O2-C11-C12 | 114.86 |  |  |
|  |  | O2-C14-C15 | 108.21 |  |  |
|  |  | C11-O2-C14 | 117.87 |  |  |
|  |  | C6-N2-C7 | 124.91 |  |  |
|  |  | N1-C5-C1 | 123.77 |  |  |
|  |  | O1-C6-C1 | 122.60 |  |  |
|  |  | N3-C7-N2 | 120.38 |  |  |
|  |  | N2-C7-S1 | 122.43 |  |  |
|  |  | C9-C8-S1 | 109.87 |  |  |
|  |  | C13-C9-N3 | 125.45 |  |  |
|  |  | O2-C11-C10 | 124.44 |  |  |

**Table S2** Radical scavenging, anti-inflammation, and anti-hyperglycemic results of compounds 2b-k

| **Samples** | **DPPH IC_50_±SD (µM)** | **ABTS IC_50_±SD (µM)** | **Inflammatory**  **IC_50_±SD (µM)** | **α-Amylase IC_50_±SD (µg)** |
| --- | --- | --- | --- | --- |
| 2b | 15.63±0.13 | 19.89±0.25 | 18.41±0.13 | 1.368±0.12 |
| 2c | 93.69±0.26 | 20.57±0.13 | 27.65±0.20 | 1.405±0.25 |
| 2d | 32.02±0.26 | 21.57±0.11 | 20.23±0.26 | 1.346±0.24 |
| 2e | 52.09±0.15 | 45.63±0.19 | 14.06±0.15 | 1.324±0.21 |
| 2f | 78.05±0.08 | 81.05±0.22 | 65.24±0.18 | 1.334±0.17 |
| 2g | 81.81±0.01 | 21.85±0.21 | 85.56±0.25 | 1.357±0.15 |
| 2h | 12.88±0.19 | 16.35±0.25 | 21.69±0.24 | 1.516±0.14 |
| 2i | 99.62±0.15 | 38.43±0.21 | 66.97±0.28 | 1.423±0.24 |
| 2j | 97.62±0.24 | 57.25±0.11 | 20.28±0.22 | 1.326±0.10 |
| 2k | 79.62±0.14 | 88.03±0.17 | 25.86±0.23 | 1.515±0.17 |
| Ascorbic  acid | 11.81±0.04 | 11.90±0.01 | - | - |
| Acarbose | - | - | - | 1.273±0.12 |
| Ketorolac | - | - | 11.79 ± 0.17 | - |

**Table S3** The inhibition efficacy of compounds 2b-k

| **DPPH Inhibition %** | | | | | | | | | | | |
| --- | --- | --- | --- | --- | --- | --- | --- | --- | --- | --- | --- |
| **Concentration (µM)** | **Ascorbic acid** | **2b** | **2c** | **2d** | **2e** | **2f** | **2g** | **2h** | **2i** | **2j** | **2k** |
| 20 | 91 | 66.2 | 32 | 36.2 | 29.2 | 35.31 | 34.94 | 84.5 | 8.89 | 34.8 | 8.89 |
| 40 | 91.8 | 86.32 | 34.9 | 56.3 | 47.1 | 38.4 | 44.35 | 85.8 | 33.34 | 37.7 | 33.34 |
| 60 | 93.7 | 87.6 | 36.1 | 80.5 | 49.2 | 41.11 | 46.41 | 86.8 | 40.65 | 43.7 | 40.65 |
| 80 | 94.3 | 88 | 48 | 87.4 | 55.8 | 43.18 | 50.02 | 87.2 | 49.83 | 46 | 49.83 |
| 100 | 96.1 | 88.4 | 51.9 | 88.3 | 59 | 45.7 | 61.07 | 87.68 | 52.22 | 50.1 | 52.31 |
| **ABTS Inhibition %** | | | | | | | | | | | |
| **Concentration (µM)** | **Ascorbic acid** | **2b** | **2c** | **2d** | **2e** | **2f** | **2g** | **2h** | **2i** | **2j** | **2k** |
| 20 | 90 | 59.6 | 58 | 56.9 | 23.4 | 27.6 | 56 | 69 | 41.6 | 28.3 | 37.6 |
| 40 | 93 | 60.8 | 59.7 | 59.6 | 27.9 | 33.8 | 57 | 71 | 44.7 | 39 | 41.8 |
| 60 | 95 | 60.9 | 59.8 | 62.5 | 29.3 | 41.5 | 58.1 | 72 | 49.5 | 42.4 | 46.3 |
| 80 | 96 | 61 | 60 | 62.6 | 35.4 | 52 | 60.2 | 72.9 | 53.9 | 45 | 47.9 |
| 100 | 96.5 | 61.4 | 60 | 63.2 | 43 | 60.1 | 62 | 76.7 | 60.4 | 47.9 | 55.8 |
| **Anti-inflammatory Inhibition %** | | | | | | | | | | | |
| **Concentration (µM)** | **Ketorolac** | **2b** | **2c** | **2d** | **2e** | **2f** | **2g** | **2h** | **2i** | **2j** | **2k** |
| 20 | 91 | 60 | 48 | 59 | 33.6 | 31 | 79 | 52 | 17.6 | 59 | 48.6 |
| 40 | 92 | 69.9 | 52.9 | 62 | 42.8 | 37 | 80.2 | 63 | 25.3 | 68 | 55 |
| 60 | 93 | 71 | 62.5 | 65.4 | 43 | 42 | 80.3 | 64.7 | 47 | 75 | 66.8 |
| 80 | 93.5 | 76 | 77 | 75.2 | 48.2 | 45.6 | 85.6 | 65 | 58 | 92.7 | 74.5 |
| 100 | 94 | 85.8 | 86 | 84.4 | 60 | 49 | 86 | 70 | 70 | 93 | 87.5 |
| **α-Amylase Inhibition %** | | | | | | | | | | | |
| **Concentration (µg)** | **Acarbose** | **2b** | **2c** | **2d** | **2e** | **2f** | **2g** | **2h** | **2i** | **2j** | **2k** |
| 2 | 85.59 | 80.52 | 79.2 | 81.69 | 86.09 | 79.2 | 80.38 | 74.08 | 77.74 | 85.94 | 74.81 |
| 4 | 83.98 | 80.38 | 74.67 | 80.52 | 77.3 | 77.89 | 73.64 | 73.2 | 76.42 | 75.4 | 74.52 |
| 6 | 82.19 | 77.01 | 73.64 | 79.2 | 74.08 | 56.22 | 73.06 | 72.47 | 74.67 | 74.67 | 69.98 |
| 8 | 80.97 | 69.1 | 62.66 | 76.13 | 72.76 | 47.87 | 40.84 | 70.57 | 71.01 | 68.96 | 69.69 |
| 10 | 80.38 | 54.9 | 57.97 | 73.64 | 56.22 | 36.74 | 29.72 | 70.13 | 70.86 | 57.1 | 55.63 |

Table S4 Single crystal results and structure refinement details of entities (2d, 2k)

|  | **Compound 2d** | **Compound 2k** |
| --- | --- | --- |
| Identification code | shelx | NA_6ET_CRYSTAL |
| Empirical formula | C_19_ H_18_ O_6_ | C_15_H_13_N_3_O_2_S |
| Weight | 343.33 g/mol | 299.34 g/mol |
| Temperature | 296(2) K | 300(2) K |
| Wavelength | 0.71073 Å | 0.71073 Å |
| Crystal system, space group | Monoclinic, P 21/c | Monoclinic, P 1 21/c 1 |
| Unit cell dimensions | a = 11.416(3) Å, α = 90°  b = 9.2671(17) Å, β = 106.674(9)°  c = 16.580(3) Å, γ = 90° | a = 7.0795(6) Å, α = 90°  b = 26.584(2) Å, β = 112.643(2)°  c = 8.0420(6) Å, γ = 90° |
| Volume | 1680.3(6) Å^3^ | 1. 1396.9(2) Å^3^ |
| Z, Calculated density | 4, 1.353 g/cm^3^ | 4, 1.423 g/cm^3^ |
| Absorption coefficient | 0.101 mm^-1^ | 0.240 mm^-1^ |
| F(000) | 720 | 624 |
| Theta range for data collection | 1.862 to 28.256° | 2.85 to 28.30° |
| Limiting indices | -15<=h<=14, -12<=k<=10, -22<=l<=18 | -9<=h<=9, 35<=k<=35, -10<=l<=10 |
| Reflections collected / unique | 13185 / 3956 [R(int) = 0.0364] | 39301/ 3479 [R(int) = 0.0651] |
| Completeness to theta | 100.0 % | 99.9% |
| Absorption correction | Semi-empirical from equivalents | Multi-Scan |
| Max. and min. transmission | 0.980 and 0.970 | 0.9740 and 0.9650 |
| Refinement method | Full-matrix least-squares on F^2^ | Full-matrix least-squares on F^2^ |
| Data / restraints / parameters | 3956 / 0 / 229 | 3479 / 0 / 191 |
| Goodness-of-fit on F^2 | 0.988 | 1.058 |
| Final R indices [I>2 sigma(I)] | R1 = 0.0688, wR2 = 0.1916 | R1 = 0.0474, wR2 = 0.0981 |
| R indices (all data) | R1 = 0.1251, wR2 = 0.2436 | R1 = 0.0929, wR2 = 0.1214 |
| Extinction coefficient | n/a | n/a |
| Largest diff. peak and hole | 0.711 and -0.360 eA^-3^ | 0.193 and -0.291 eÅ^-3^ |

**Table S5** molecular docking grid configuration parameters

| Ligand | Spacing (Å) | Center | | | npts | | |
| --- | --- | --- | --- | --- | --- | --- | --- |
| 2b | 0.492 | -7.5677 | 13.893 | -19.025 | 78 | 86 | 88 |
| 2c | 0.375 | -3.542 | 7.126 | -19.025 | 108 | 90 | 86 |
| 2d | 0.480 | -8.182 | 13.971 | -19.025 | 66 | 102 | 88 |
| 2e | 0.375 | -8.182 | 8.384 | -19.025 | 74 | 86 | 96 |
| 2f | 0.375 | -9.6 | 13.527 | -18.718 | 88 | 104 | 84 |
| 2g | 0.375 | -9.6 | 13.527 | -18.718 | 88 | 104 | 84 |
| 2h | 0.480 | -8.182 | 13.971 | -19.025 | 66 | 102 | 88 |
| 2i | 0.631 | -7.683 | 13.446 | -21.135 | 50 | 66 | 46 |
| 2j | 0.436 | -8.182 | 11.719 | -19.025 | 102 | 90 | 92 |
| 2k | 0.402 | -7.124 | 9.057 | -13.296 | 72 | 66 | 82 |

**Table S6** MTT assay OD values for varying concentrations

| Compound code | Concentration (µM) | Triplicate values | | | Average OD |
| --- | --- | --- | --- | --- | --- |
|  |  | OD1 | OD2 | OD3 |  |
| Control |  | 0.922 | 0.927 | 0.924 | 0.924 |
| Compound 2b | 10 | 0.790 | 0.792 | 0.785 | 0.789 |
|  | 20 | 0.719 | 0.712 | 0.711 | 0.714 |
|  | 30 | 0.634 | 0.629 | 0.636 | 0.633 |
|  | 40 | 0.542 | 0.541 | 0.546 | 0.543 |
|  | 50 | 0.418 | 0.416 | 0.423 | 0.419 |
| Compound 2c | 10 | 0.824 | 0.826 | 0.819 | 0.823 |
|  | 20 | 0.754 | 0.753 | 0.758 | 0.755 |
|  | 30 | 0.689 | 0.695 | 0.692 | 0.692 |
|  | 40 | 0.596 | 0.604 | 0.606 | 0.602 |
|  | 50 | 0.520 | 0.525 | 0.518 | 0.521 |

**Table S7** Toxicity evaluation of synthesized compounds

| **Conjugate Concentration (µM)** | **% of Cell viability** | |
| --- | --- | --- |
|  | **Compound 2b** | **Compound 2e** |
| Control | 100 | 100 |
| 10 | 85.43 | 89.1 |
| 20 | 77.36 | 81.67 |
| 30 | 68.52 | 74.84 |
| 40 | 58.79 | 65.12 |
| 50 | 45.33 | 56.39 |


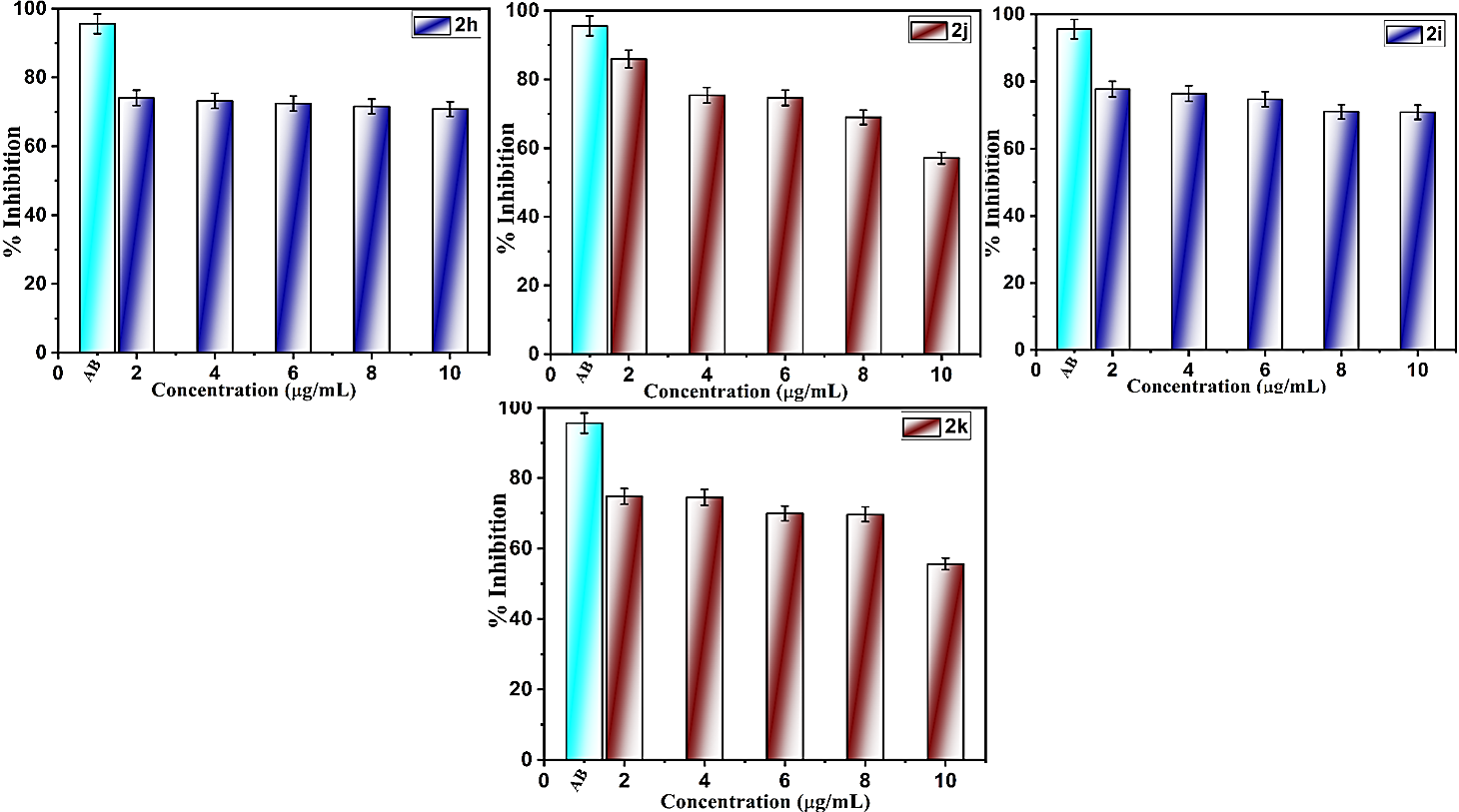

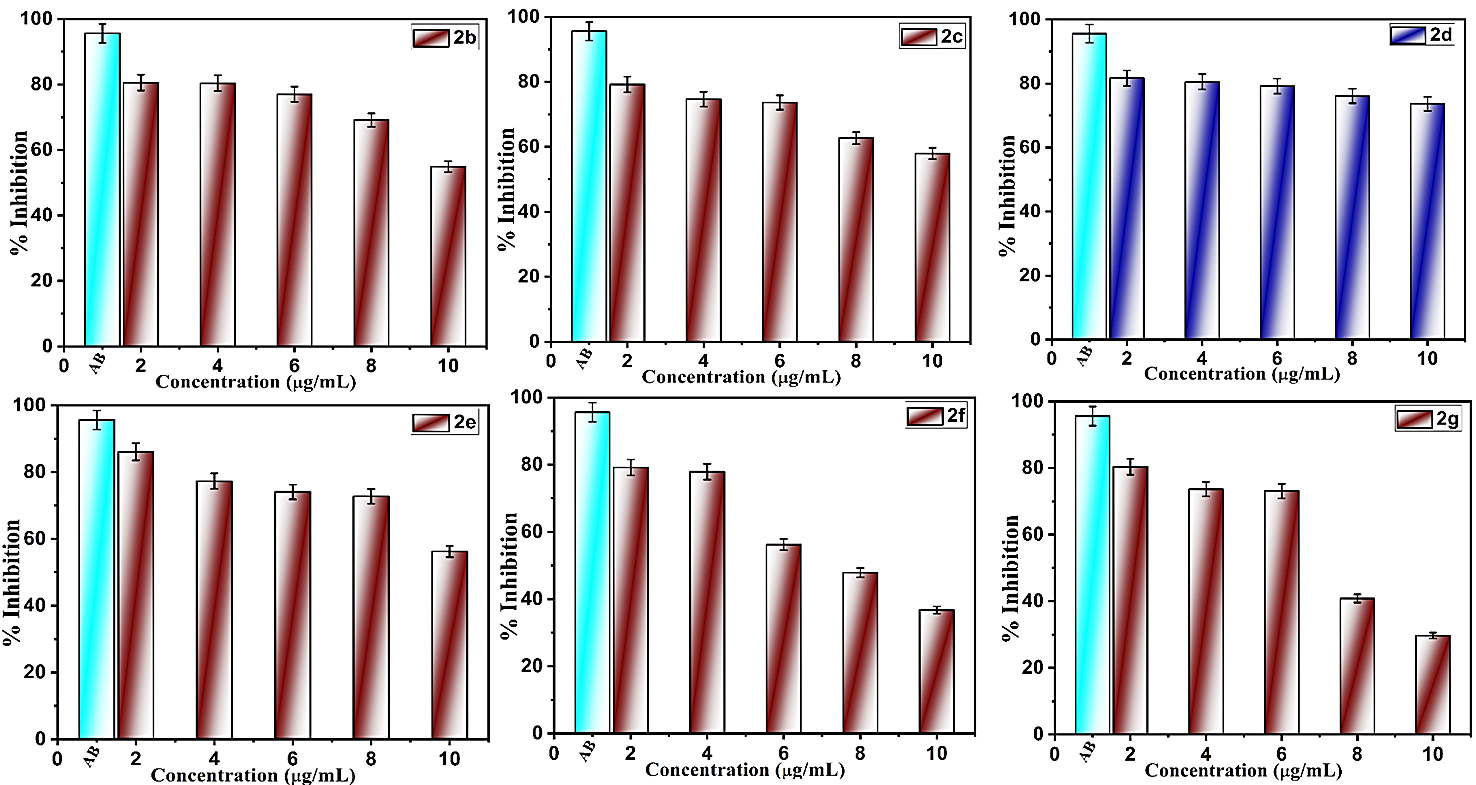


**Figure S41.** Inhibitory activity of compounds 2b-k against α-amylase enzyme


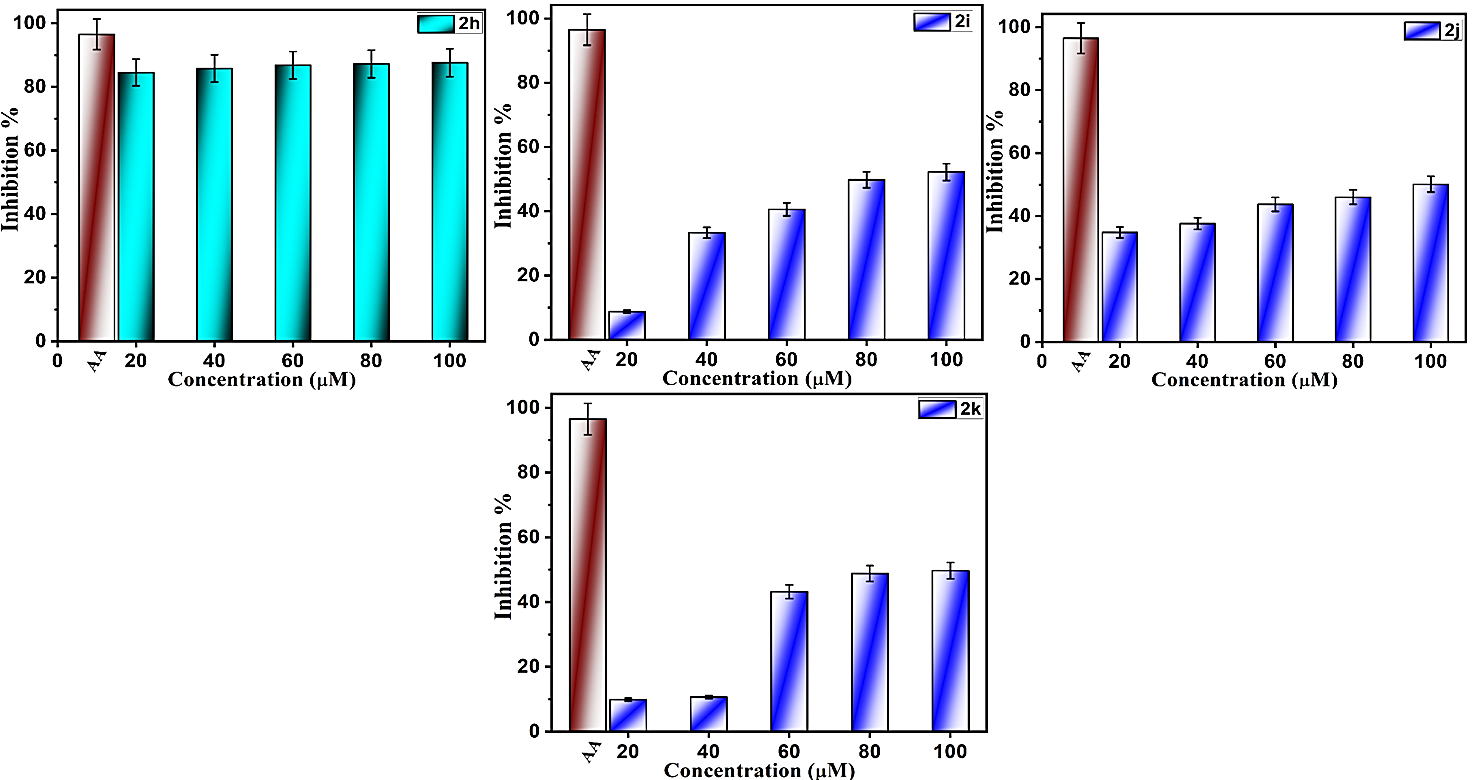

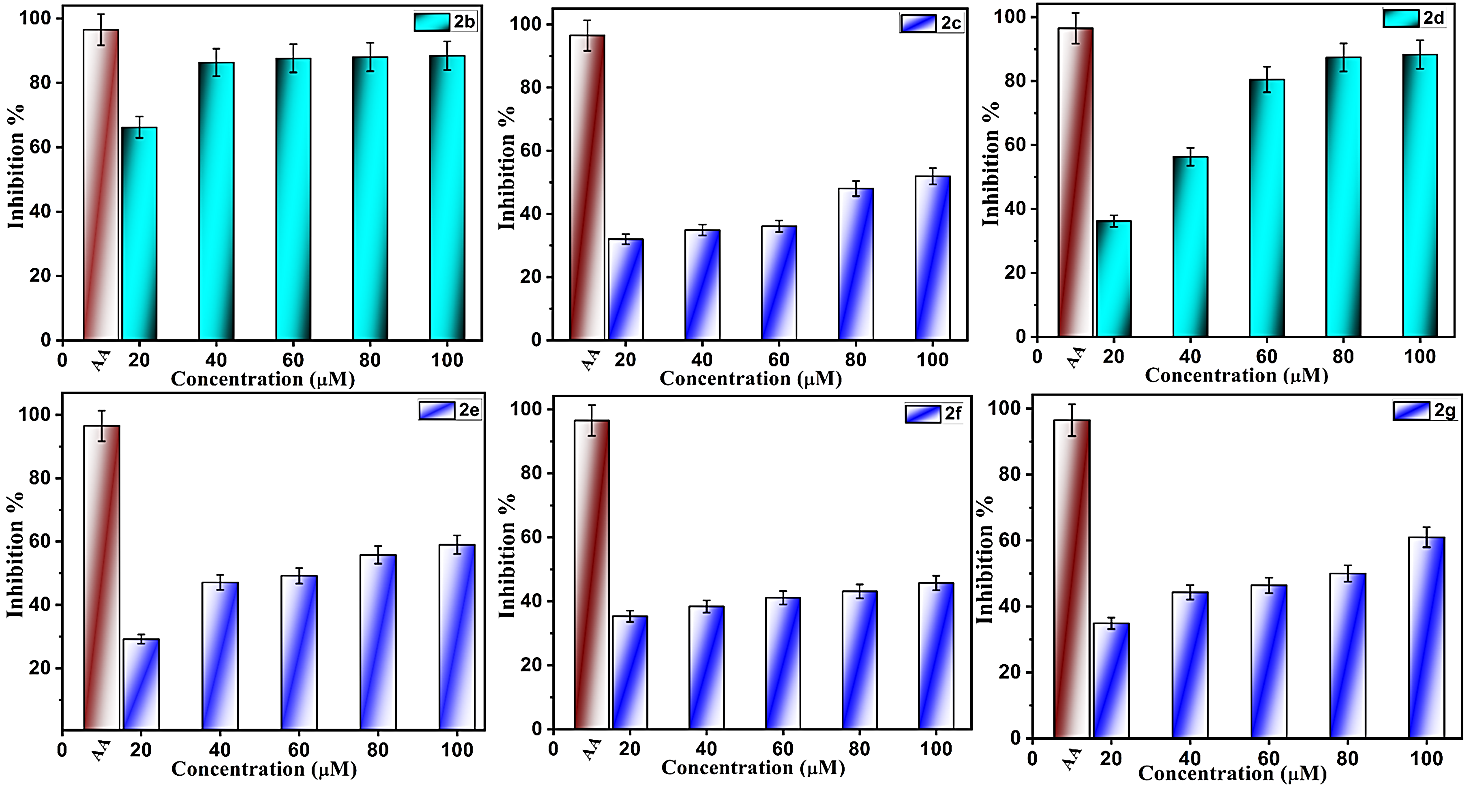


**Figure S42.** Inhibition evaluation of compounds 2b-k against DPPH


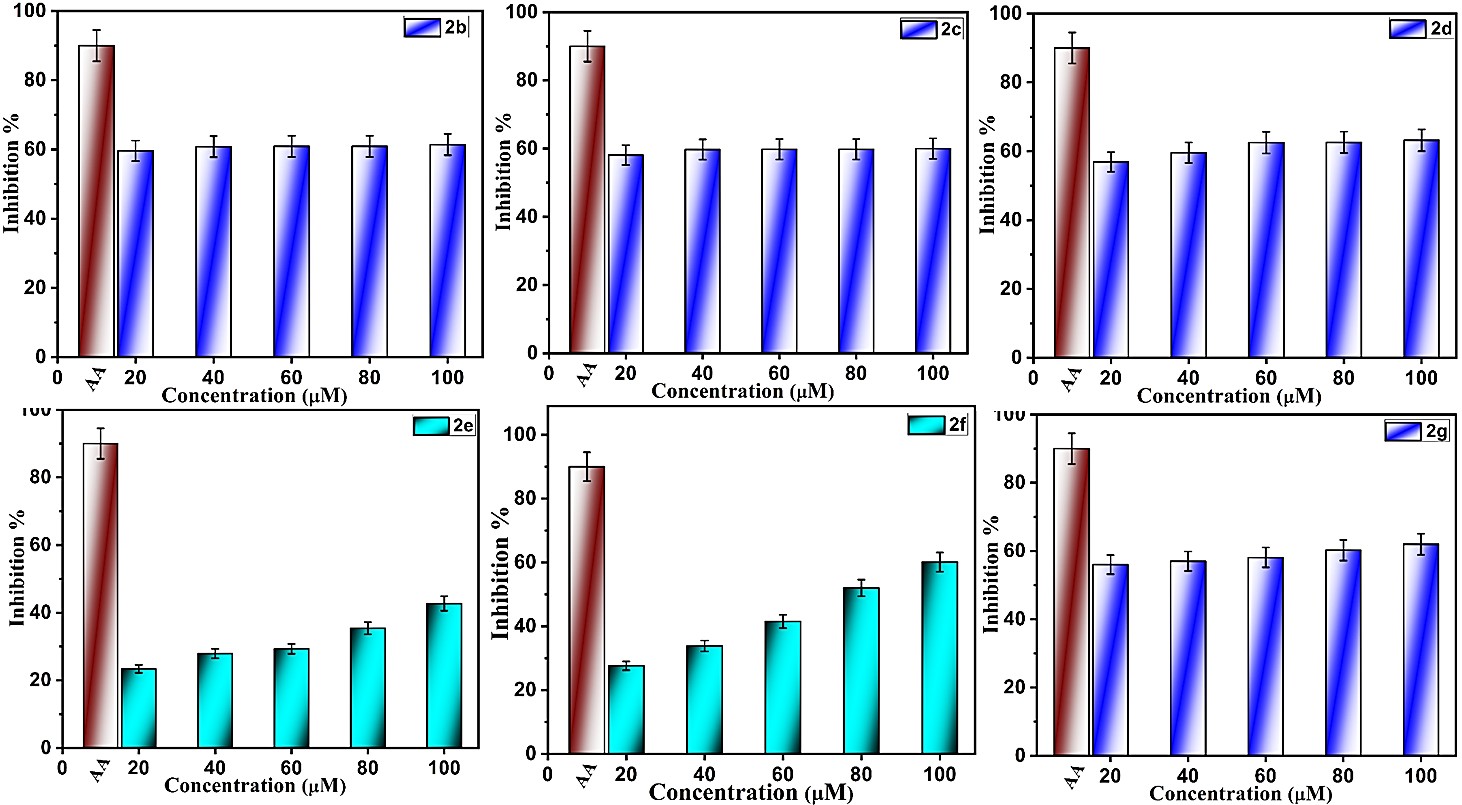

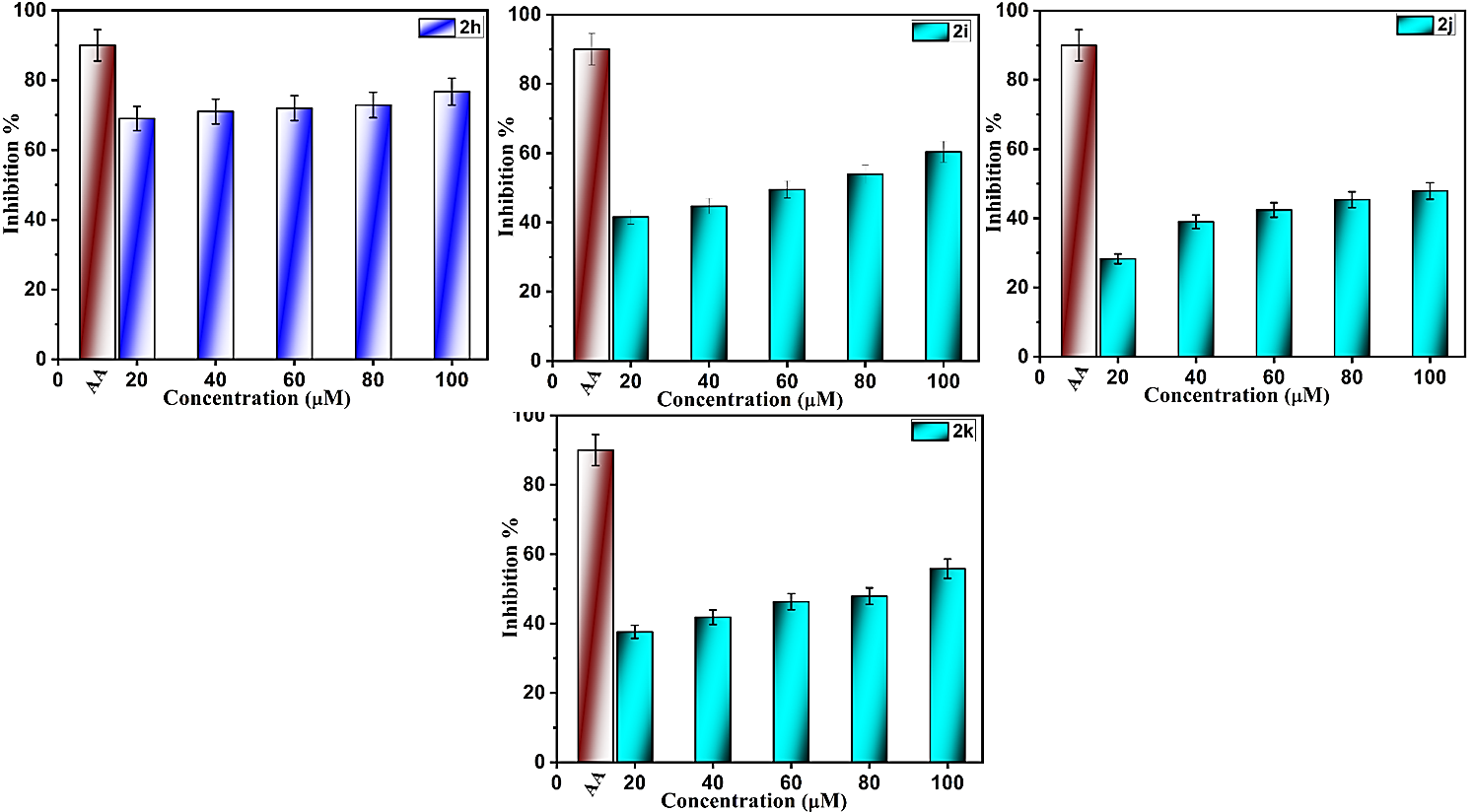


**Figure S43.** Inhibition evaluation of compounds 2b-k against ABTS


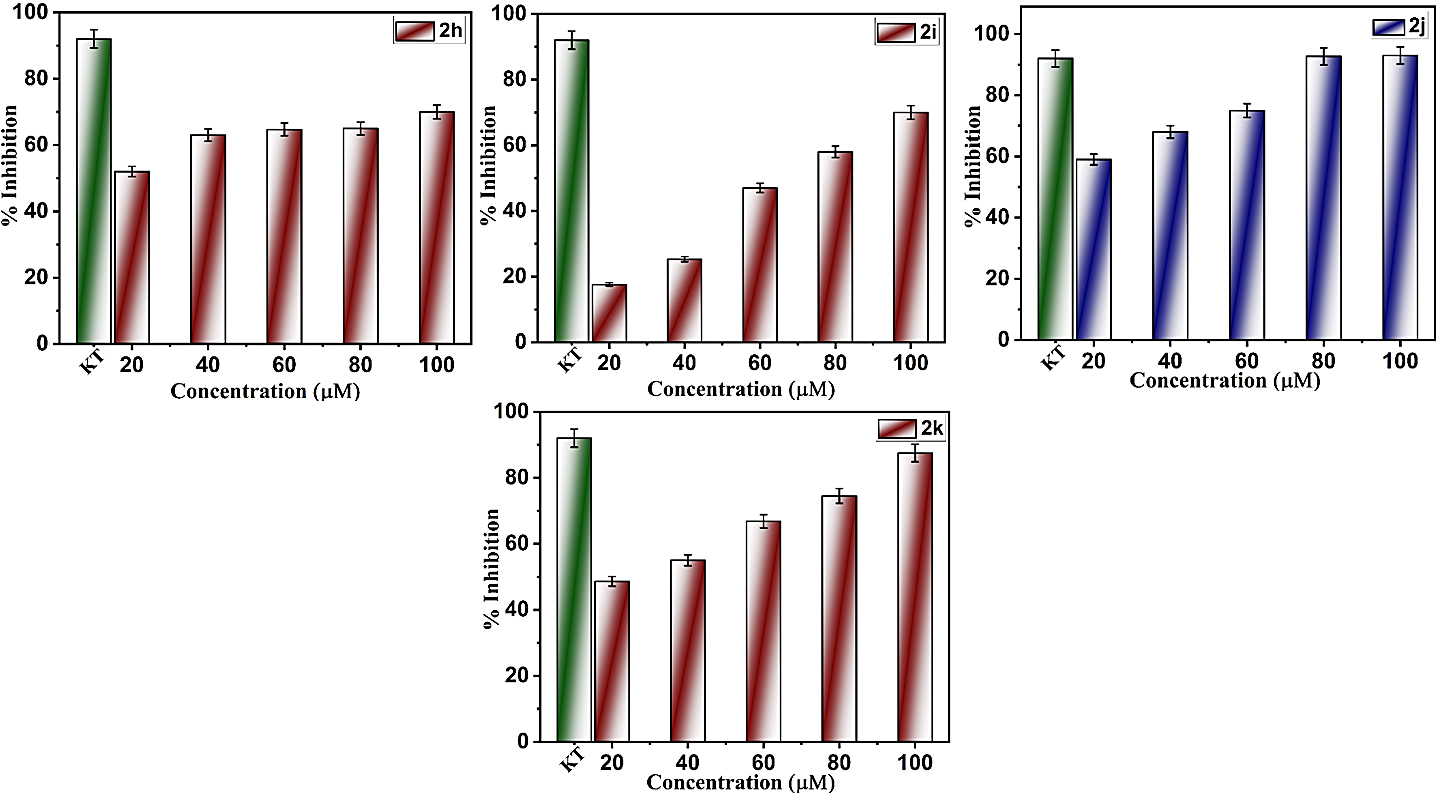

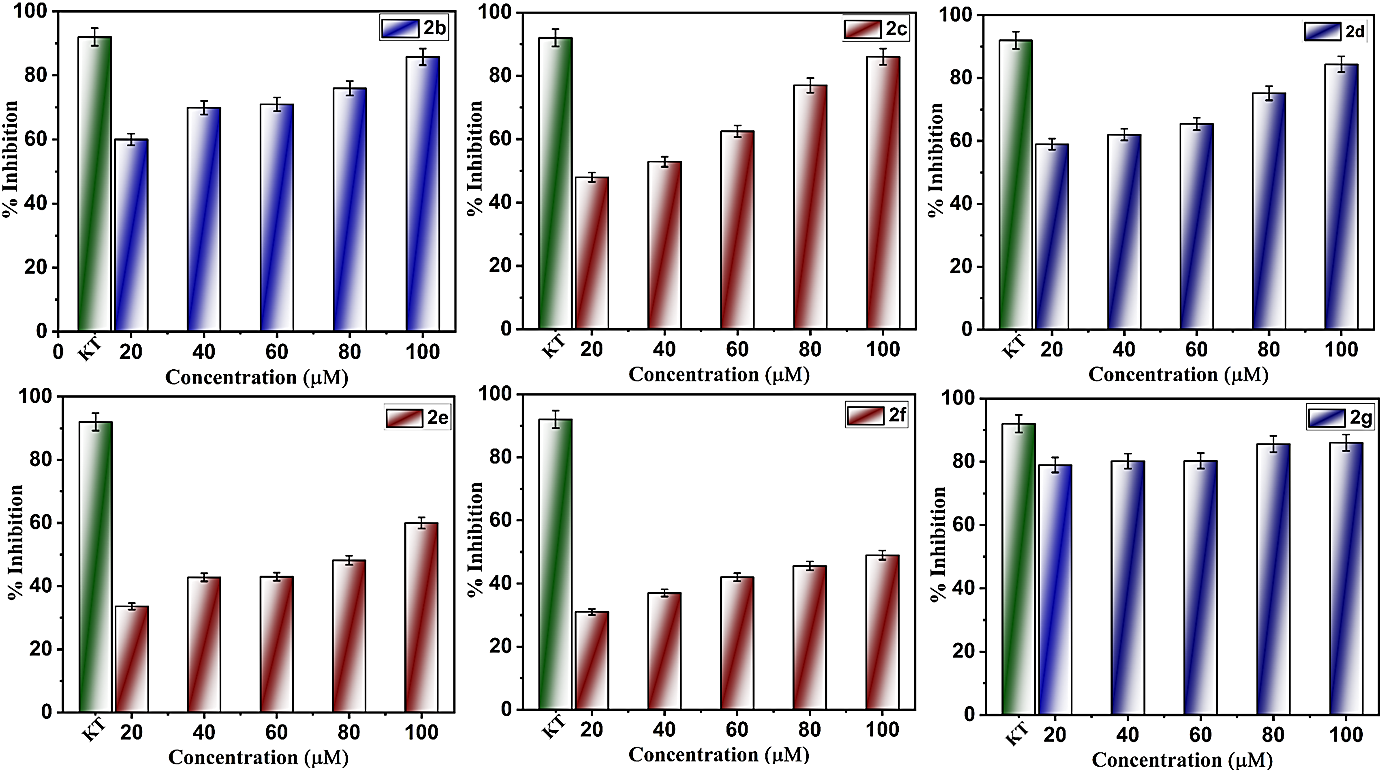


Figure S44. Evaluation of anti-inflammatory action of compounds 2b-k

Figure S45. *In-silico* Docking analysis of compounds 2b-k interactions were visualized by the Discovery Studio R2 software
